# Cleavage-Polyadenylation Factor Cft1 and SPX Domain Proteins Are Agents of Inositol Pyrophosphate Toxicosis in Fission Yeast

**DOI:** 10.1128/mbio.03476-21

**Published:** 2022-01-11

**Authors:** Beate Schwer, Angad Garg, Ana M. Sanchez, Mindy A. Bernstein, Bradley Benjamin, Stewart Shuman

**Affiliations:** a Department of Microbiology and Immunology, Weill Cornell Medical College, New York, New York, USA; b Molecular Biology Program, Sloan-Kettering Institute, New York, New York, USA; c Gerstner Sloan Kettering Graduate School of Biomedical Sciences, New York, New York, USA; Harvard Medical School

**Keywords:** SPX domain, inositol pyrophosphate, phosphate homeostasis, transcription termination

## Abstract

Inositol pyrophosphate (IPP) dynamics govern expression of the fission yeast phosphate homeostasis regulon via their effects on lncRNA-mediated transcription interference. The growth defects (ranging from sickness to lethality) elicited by fission yeast mutations that inactivate IPP pyrophosphatase enzymes are exerted via the agonistic effects of too much 1,5-IP8 on RNA 3′-processing and transcription termination. To illuminate determinants of IPP toxicosis, we conducted a genetic screen for spontaneous mutations that suppressed the sickness of Asp1 pyrophosphatase mutants. We identified a missense mutation, C823R, in the essential Cft1 subunit of the cleavage and polyadenylation factor complex that suppresses even lethal Asp1 IPP pyrophosphatase mutations, thereby fortifying the case for 3′-processing/termination as the target of IPP toxicity. The suppressor screen also identified Gde1 and Spx1 (SPAC6B12.07c), both of which have an IPP-binding SPX domain and both of which are required for lethality elicited by Asp1 mutations. A survey of other SPX proteins in the proteome identified the Vtc4 and Vtc2 subunits of the vacuolar polyphosphate polymerase as additional agents of IPP toxicosis. Gde1, Spx1, and Vtc4 contain enzymatic modules (glycerophosphodiesterase, RING finger ubiquitin ligase, and polyphosphate polymerase, respectively) fused to their IPP-sensing SPX domains. Structure-guided mutagenesis of the IPP-binding sites and the catalytic domains of Gde1 and Spx1 indicated that both modules are necessary to elicit IPP toxicity. Whereas Vtc4 polymerase catalytic activity is required for IPP toxicity, its IPP-binding site is not. Epistasis analysis, transcriptome profiling, and assays of Pho1 expression implicate Spx1 as a transducer of IP8 signaling to the 3′-processing/transcription termination machinery.

## INTRODUCTION

The inositol pyrophosphates (IPPs) 5-IP7, 1-IP7, and 1,5-IP8 are generated from phytic acid (IP6) by the action of IPP kinases (1, 2). Cellular IPP dynamics are dictated by a balance between these kinases and several IPP pyrophosphatase enzymes that remove the IPP β-phosphate groups. IPPs play central roles in eukaryal cellular phosphate homeostasis (3–7), a transcriptional response to phosphate limitation entailing the upregulation of genes involved in phosphate acquisition. In the fission yeast Schizosaccharomyces pombe, three genes—*pho1* (cell surface acid phosphatase), *pho84* (inorganic phosphate transmembrane transporter), and *tgp1* (glycerophosphate transporter)—constitute a phosphate (*PHO*) regulon (8). The *PHO* genes are actively repressed under phosphate-replete conditions and derepressed during phosphate starvation. Repression in phosphate-replete cells is achieved via tandem transcriptional interference with the *pho1*, *pho84*, and *tgp1* mRNA promoters, mediated by synthesis in *cis* of upstream long noncoding RNAs (lncRNAs): *prt*, *prt2*, and *nc-tgp1*, respectively (9). *pho1* is derepressed under phosphate-replete conditions by a variety of genetic maneuvers that favor “precocious” *prt* lncRNA 3′-processing/termination in response to poly(A) signals upstream of the *pho1* promoter (10, 11). *prt* lncRNA termination is particularly sensitive to changes in IPP dynamics (12).

The initial hints that IPPs affect fission yeast *PHO* expression emerged from findings by the Wykoff lab that a deletion of Asp1 (an IPP kinase) hyperrepressed *pho1* and a deletion of Aps1 (a Nudix-family IPP pyrophosphatase [13]) derepressed *pho1* under phosphate-replete conditions (14, 15). Asp1 consists of an N-terminal kinase domain that phosphorylates 5-IP7 to 1,5-IP8 and a C-terminal pyrophosphatase domain that hydrolyzes 1,5-IP8 back to 5-IP7 (16, 17). Deleting Asp1, or a kinase-inactivating Asp1-D333A mutation, eliminates intracellular IP8 and increases the level of 5-IP7, whereas a pyrophosphatase-dead Asp1-H397A mutation elevates intracellular IP8 (16, 17).

IPPs control fission yeast phosphate homeostasis via the 3′-processing/termination machinery (12). Increasing IP8 via Asp1-H397A derepresses the *PHO* mRNAs (and leads to precocious *prt* lncRNA termination) in a manner that depends genetically on the cleavage and polyadenylation factor complex (CPF) and the transcription termination factor Rhn1. Tandem inactivation of the IPP pyrophosphatases Asp1 and Aps1 is synthetically lethal, indicating that too much IP8 is toxic to fission yeast. A key finding was that mutations of CPF subunits suppressed the lethality of *asp1-H397A aps1*Δ (12). Absence of IP8 in *asp1*Δ and *asp1-D333A* cells resulted in *pho1* hyperrepression. The observation that *asp1*Δ (no IP8) was synthetically lethal with CPF subunit mutations argued that IP8 has an important, albeit genetically redundant, role to play in essential 3′-processing/termination events (12). These results established a novel action for IPPs as agonists of Pol2 transcription termination.

lncRNA control of *PHO* gene expression is affected by the Thr4 phosphosite of the RNA polymerase II (Pol2) carboxy-terminal domain (CTD), pan-alanine mutation of which results in *pho1* and *pho84* hyperrepression (10, 18). A genetic screen for mutations that derepress Pho1 acid phosphatase expression in *CTD-T4A* cells (19) yielded 18 independent *STF* (suppressor of threonine four) isolates, each of which bore a mutation in the Asp1 pyrophosphatase domain. Focused characterization of two isolates, *STF-6* and *STF-9* strains, identified 51 protein-coding transcripts (including the *pho1*, *pho84*, and *tgp1* mRNAs) that were upregulated versus the *T4A* parental strain. The *STF-6* and *STF-9* alleles—*asp1-386*(*Stop*) and *asp1-493*(*Stop*), respectively—were lethal in a wild-type Pol2-CTD background. However, viability was restored when *STF-6* and *STF-9* were combined with mutations of CPF subunits or Rhn1, in which context Pho1 was also derepressed (19). These results implicated Asp1 pyrophosphatase activity in restraining IP8 synthesis by Asp1 kinase, without which IP8 could attain toxic levels that drive overzealous CPF/Rhn1-dependent termination.

**FIG 1:**
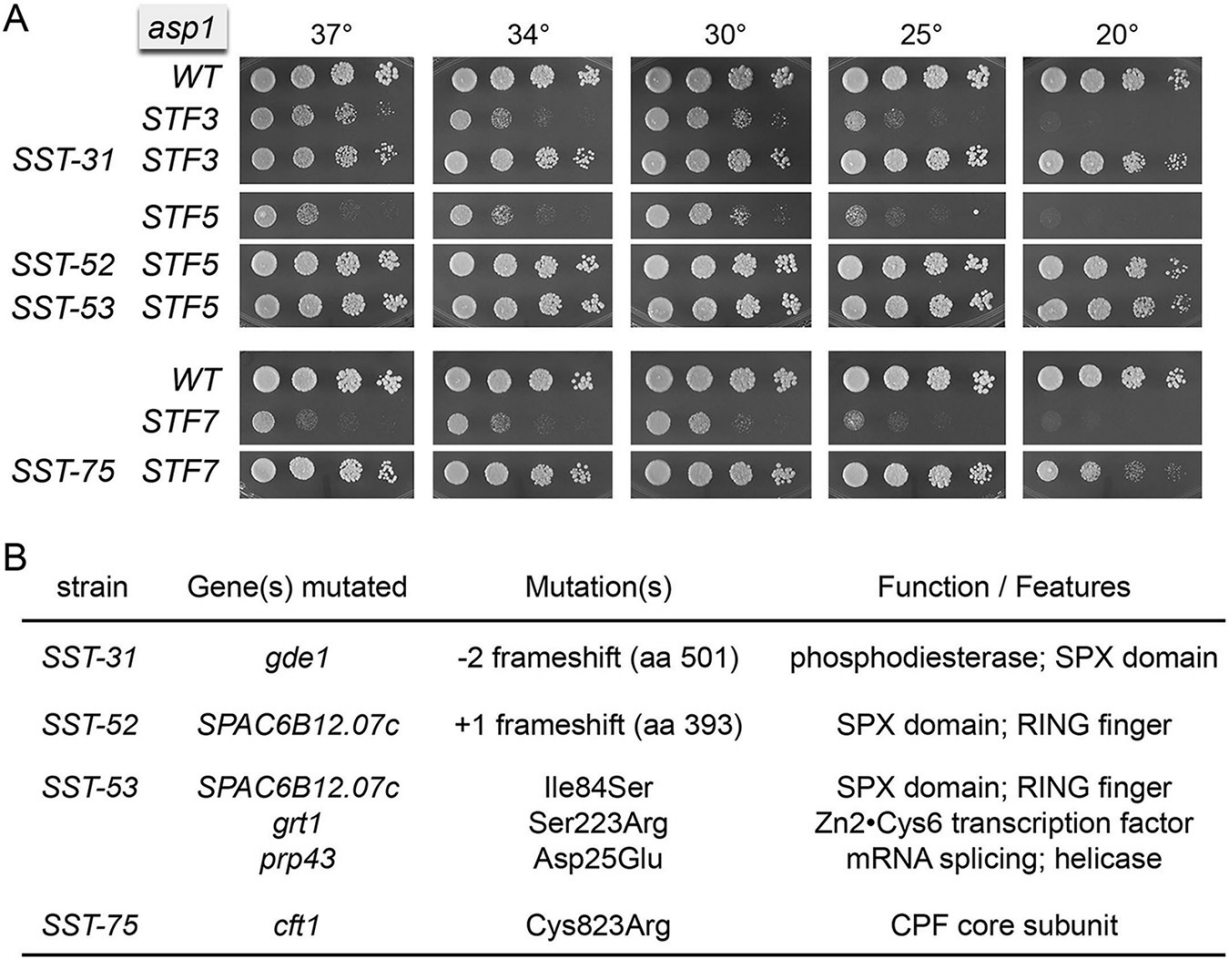
Fission yeast *SST* mutants that suppress *STF* alleles of Asp1. (A) Serial 5-fold dilutions of fission yeast strains (as specified on the left) were spot tested for growth on YES agar at the indicated temperatures. (B) Whole-genome sequencing of *SST* strains revealed the indicated genetic mutations.

The mechanism of “IPP toxicosis” and the signaling pathway that connects IP8 elevation to Pol2 termination are unknown. To close this knowledge gap, we conducted here a forward genetic screen for spontaneous mutations that suppressed the toxicity of IPP pyrophosphatase mutations. For this purpose, we exploited three *STF* alleles of *asp1*—*STF-3* (*G863D*), *STF-5* (*C643Y*), and *STF-7* (*H686Y*)—that caused severe growth defects in a wild-type CTD background (Fig. 1A). Via this *SST* (suppressor of suppressor of Thr4) screen, we identified a missense mutation of the essential Cft1 subunit of CPF that sufficed to suppress even the lethal IPP pyrophosphatase mutations *STF-6* and *STF-9.* This result fortifies the case for CPF as a target of IPP toxicosis.

The *SST* screen also identified two new agents of fission yeast IPP transactions, Gde1 and Spx1 (SPAC6B12.07c), both of which have a predicted IPP-binding SPX domain (4, 20) and both of which are required for IP8 toxicity elicited by Asp1 pyrophosphatase mutations. Gde1 is a large protein composed of an N-terminal SPX domain, a central ankyrin repeat domain, and a C-terminal glycerophosphodiesterase catalytic domain. Spx1 consists of an N-terminal SPX module and a C-terminal ubiquitin ligase RING finger module. Whereas deletion of Gde1 has but a modest effect on *pho1* expression, deletion of Spx1 hyperrepresses *pho1* expression, erases *pho1* derepression by Asp1 pyrophosphatase mutations, and attenuates *pho1* induction in response to phosphate starvation. In light of the results of the *SST* screen, we queried whether any of the four other fission yeast SPX proteins contribute to IPP toxicity and found that deletions of the Vtc2 and Vtc4 subunits of the vacuolar polyphosphate polymerase complex (21) suppressed lethal IPP pyrophosphatase mutations. Alanine mutations of the IPP-binding sites of Spx1 and Gde1 phenocopied *spx1*Δ and *gde1*Δ with respect to relief of IPP toxicity, as did alanine mutations of the Spx1 ubiquitin ligase and Gde1 glycerophosphodiesterase catalytic domains. Whereas mutation of the Vtc4 polyphosphate polymerase active site phenocopied *vtc4*Δ, mutation of the IPP-binding site of Vtc4 did not. Epistasis analysis implicates Spx1 as a transducer of IP8 signaling to the 3′-processing/transcription termination machinery.

## RESULTS

### Isolation of spontaneous suppressors of IPP toxicosis.

*STF-3* (*G863D*), *STF-5* (*C643Y*), and *STF-7* (*H686Y*) pyrophosphatase mutations of *asp1* cause a severe growth defect at all temperatures in a wild-type genetic background (Fig. 1A). We screened for candidate *SST* (suppressors of STF) mutants by plating *STF-3*, *STF-5*, and *STF-7* cells on YES (yeast extract with supplement) agar at 30°C and selecting rare single colonies that grew to large size against a background of tiny colonies. These were grown and restreaked for single colonies, which were homogeneously larger than the colonies of the respective parental *STF* strains. Four *SST STF* isolates were selected for further analysis after spot testing for growth in parallel with wild-type and parental *STF* controls. The *SST-52 STF5* and *SST-53 STF5* strains grew as well as the wild-type at all temperatures, as gauged by colony number and size (Fig. 1A). *SST-75 STF7* cells grew well at 25°C to 37°C but were slower growing than the wild-type at 20°C. *SST-31 STF3* cells displayed a small-colony phenotype at 37°C (Fig. 1A). To rule out the possibility that the *STF* suppression resulted from reversion of the original *asp1-STF* missense mutation or from a kinase-inactivating mutation in the N-terminal IPP kinase domain of Asp1, we amplified and sequenced the *asp1* open reading frame (ORF) from the four *SST STF* isolates and verified that the original *STF* alleles were unchanged.

### Identification of IPP toxicosis suppressor mutations by whole-genome sequencing.

Paired-end Illumina sequencing of unamplified genomic DNA from the four *SST STF* strains was performed to achieve at least 100-fold coverage of each fission yeast genome. The *SST STF* genomes were compared to those of the respective *STF* strains that we sequenced previously (19). The mutations found in each of the four *SST* strains are listed in Fig. 1B. The *SST*-associated single lesions in *SST-75*, *SST-31*, and *SST-52* cells map to three protein-coding genes: *cft1*, *gde1*, and *SPAC6B12.07c*, respectively. *SST-53* cells have a different lesion in the *SPAC6B12.07c* gene as well as missense mutations in *grt1* and *prp43* (Fig. 1B). Given the apparent identical growth of the *SST-52 STF5* and *SST-53 STF5* strains with mutations in the *SPAC6B12.07c* gene, we suppose that the *grt1* and *prp43* missense changes are not germane to the *SST-53* suppressor phenotype.

### Suppression of IPP toxicity by Cft1 mutation.

**FIG 2:**
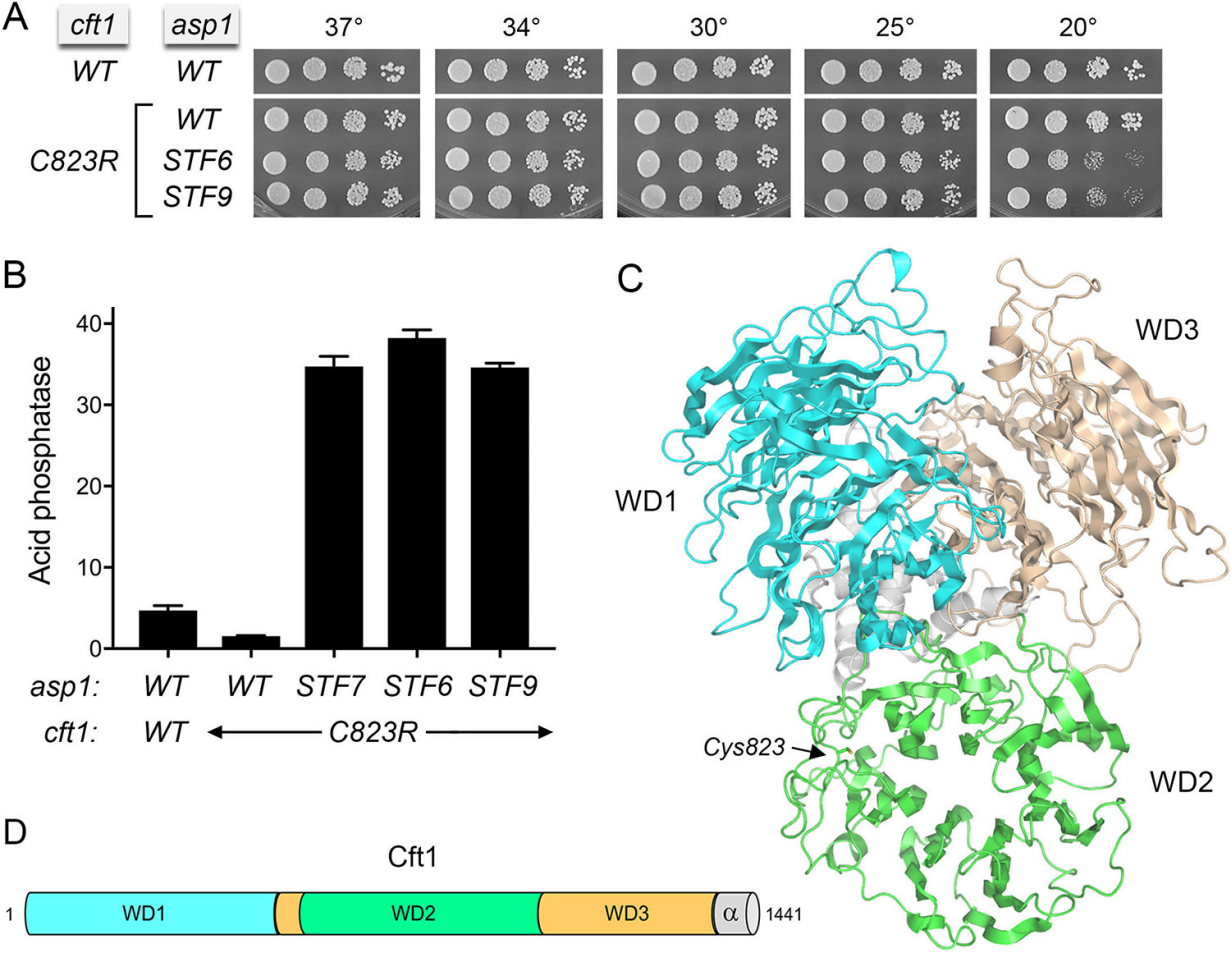
Suppression of IPP toxicity by Cft1 mutation. (A) Fission yeast strains with the indicated *asp1* and *cft1* alleles were spot tested for growth on YES agar at the indicated temperatures. (B) Cells with the indicated *asp1* and *cft1* alleles were grown to an *A*_600_ of 0.5 to 0.8 in liquid culture in YES medium at 30°C. Cells were then harvested, washed with water, and assayed for Pho1 acid phosphatase activity by conversion of *p*-nitrophenylphosphate to *p*-nitrophenol. Activity is expressed as the ratio of *A*_410_ (*p*-nitrophenol production) to *A*_600_ (input cells). (C) Phyre2 model of fission yeast Cft1 tertiary structure, colored by domain as shown in panel D. The Cys823 residue that is mutated in the SST-75 suppressor allele is located in the WD2 domain and depicted as a stick model. (D) Cartoon model of Cft1 domain organization.

*cft1* is an essential gene encoding a 1,441-amino-acid (aa) subunit of fission yeast CPF, a 13-subunit protein assembly responsible for the cotranscriptional 3′-cleavage and 3′-polyadenylation of Pol2 transcripts that precedes Pol2 transcription termination (22). The *SST-75* allele of *cft1* replaces Cys823 with arginine. To gain insight into the possible impact of this mutation, we submitted the Cft1 amino acid sequence to the Phyre2 structure modeling server (23), which returned a “top hit” tertiary structure model (Fig. 2C) templated on the cryo-EM structure of the Saccharomyces cerevisiae homolog (PDB ID 6EOJ) (24). Cft1 consists of three WD repeat beta propeller domains and a C-terminal α-helical module (Fig. 2C and D). The C823R mutation resides within the WD2 domain. Cys823 points into the hydrophobic core of WD2, and it is likely that an Arg mutation would perturb the local structure.

Our presumption was that *cft1-C823R* is a hypomorphic mutation affecting 3′-processing by CPF, which ameliorates the precocious 3′-processing/termination that underlies the slow growth of the parental *STF7* strain. Accordingly, we aimed to gauge (i) whether *cft1-C823R* elicits a phenotype in a wild-type genetic background, and (ii) whether *cft1-C823R* could suppress the lethality of the *STF6* and *STF9* mutations of the Asp1 pyrophosphatase (19). The *SST-75 STF7* strain was crossed to an *asp1-WT* strain in which the *asp1* locus was marked with a flanking drug resistance cassette. Viable *cft1-C823R* haploid progeny were recovered after sporulation, selection for the *asp1-WT* locus, and genotyping *cft1-C823R* by PCR amplification and sequencing the relevant segment of the *cft1* ORF. The *cft1-C823R* cells grew as well as the wild-type on YES agar at all temperatures tested (Fig. 2A).

*cft1-C823R* cells were mated to *STF6 rpb1-CTD-T4A* and *STF9 rpb1-CTD-T4A* strains differentially marked at the *asp1* and *rpb1-CTD-T4A* loci. Random spores were screened for the various markers, and viable *cft1-C823R STF6* and *cft1-C823R STF9* haploid progeny were recovered at the expected frequencies. The *cft1-C823R STF6* and *cft1-C823R STF9* strains grew as well as wild-type cells on YES agar at 25 to 37°C but displayed a cold-sensitive growth defect at 20°C (Fig. 2A).

To determine how *cft1-C823R* impacts *pho1* expression under phosphate-replete conditions (a sensitive gauge of mutational effects on lncRNA transcriptional interference), we assayed the wild-type and mutant strains for Pho1 acid phosphatase activity. The *cft1-C823R* allele elicited a 3-fold hyperrepression of Pho1 expression vis-à-vis wild-type *cft1^+^* (Fig. 2B), presumably because CPF containing Cft1-C823R is less effective in terminating *prt* lncRNA transcription prior to Pol2 traversing the *pho1* mRNA promoter. In contrast, Pho1 was derepressed by 7- to 8-fold in *cft1-C823R STF9* and *cft1-C823R STF6* cells and to a similar extent in *cft1-C823R STF7* cells (the original context in which *cft1-C823R* was identified) (Fig. 2B). The acid phosphatase activity of *cft1-C823R STF7* cells (level of 35) was ∼3-fold lower than that of *STF7* cells (level of 110 [19]), signifying that the *cft1-C823R* allele only partially reversed the effect of *STF7* on *PHO* gene expression.

### Two SPX proteins are implicated in IPP toxicosis.

The *gde1* and *SPAC6B12.07c* genes identified in the *SST* screen have no obvious connection to 3′-processing/termination, but they do share one potentially instructive property: they encode proteins with a predicted SPX domain. The SPX domain adopts a distinctive tertiary structure composed of a three-helix bundle and an N-terminal α-helix hairpin (4, 5, 20). The SPX domain can exist as a stand-alone polypeptide or be fused to a variety of flanking protein domains that have known or imputed functions. SPX domains bind, and are thought to act as sensors for, IPP signaling molecules. A conserved constellation of tyrosine and lysine side chains in SPX proteins comprise the high-affinity binding site for IPP ligands. Thus, the finding that mutations in two different SPX-domain proteins ameliorate IPP toxicosis in fission yeast unveils them as key IPP sensors.

Gde1 is a large protein that is inessential for fission yeast vegetative growth. The Gde1 protein is (to our inspection) misannotated in Pombase as a 1,076-aa polypeptide that lacks extra N-terminal peptide sequence (∼50 aa with a basic patch) that defines the SPX domain. The missing N-terminal segment is present in the Gde1 orthologs from the fission yeast species Schizosaccharomyces cryophilus and Schizosaccharomyces octosporus. Conceptual translation of the DNA sequence preceding the annotated Met translation start codon revealed a continuous open reading frame, initiating with a Leu codon and specifying a 58-aa peptide that is highly conserved with respect to the equivalent N-terminal segments of the two other fission yeast Gde1 proteins (see Fig. S1A in the supplemental material). The corrected 1134-aa Gde1 polypeptide is shown in Fig. S1B. Analysis of fission yeast Gde1 in Phyre2 predicts that it consists of an N-terminal 182-aa SPX domain (shaded blue in Fig. S1B) and a 332-aa C-terminal glycerophosphodiesterase catalytic domain (for which the protein is named) (shaded yellow in Fig. S1B). Phyre2 predicts that the Gde1 segment spanning from the end of the SPX domain to approximately residue 525 comprises a series of tandem ankyrin repeats. The Gde1 catalytic domain belongs to a widely distributed glycerophosphodiesterase enzyme family with a conserved tertiary structure and active site for metal-dependent hydrolysis to form glycerol-3-phosphate and an R-OH leaving group (25, 26). The budding yeast Gde1 homolog is a key player (along with budding yeast glycerophosphodiester transporter Git1, the homolog of S. pombe Tgp1) in the metabolism of glycerophosphodiesters produced via deacylation of phospholipids. Specifically, yeast Gde1 is an intracellular enzyme that acts on glycerophosphoinositol or glycerophosphocholine imported by Git1/Tgp1 to generate inositol or choline and glycerol-3-phosphate, thereby providing a potential source of phosphate during periods of nutrient starvation (27). The *SST-31* mutation in *gde1* is a −2 frameshift at the codon for aa 501 that results in translation of a foreign heptapeptide following Val500 and truncation of Gde1 thereafter in response to a new in-frame stop codon (Fig. S1C). Thus, the *SST-31* allele eliminates the catalytic domain of Gde1.

10.1128/mbio.03476-21.1 FIG S1 S. pombe Gde1. (A) The methionine start site for S. pombe Gde1 misannotated in Pombase (underlined) is preceded by an open reading frame encoding a 58-aa N-terminal leader peptide, starting at a Leu codon, that is highly conserved with the aligned N-terminal segments of Gde1 orthologs from *S. cryophilus* and S. octosporus. Positions of amino acid side chain identity/similarity in all three Gde1 proteins preceding the underlined methionine are indicated by dots above the alignment. (B) Corrected amino acid sequence of the Gde1 polypeptide. The N-terminal SPX domain and the C-terminal glycerophosphodiesterase catalytic domain are shaded cyan and yellow, respectively. The arginine codon suffering a −2 frameshift in the *SST-31* strain is highlighted in green. (C) The *SST-31* frameshift results in translation of a foreign heptapeptide and subsequent truncation of Gde1 at a new stop codon. Download FIG S1, JPG file, 0.8 MB. Copyright © 2022 Schwer et al. 2022 Schwer et al. https://creativecommons.org/licenses/by/4.0/ This content is distributed under the terms of the Creative Commons Attribution 4.0 International license.

**FIG 3:**
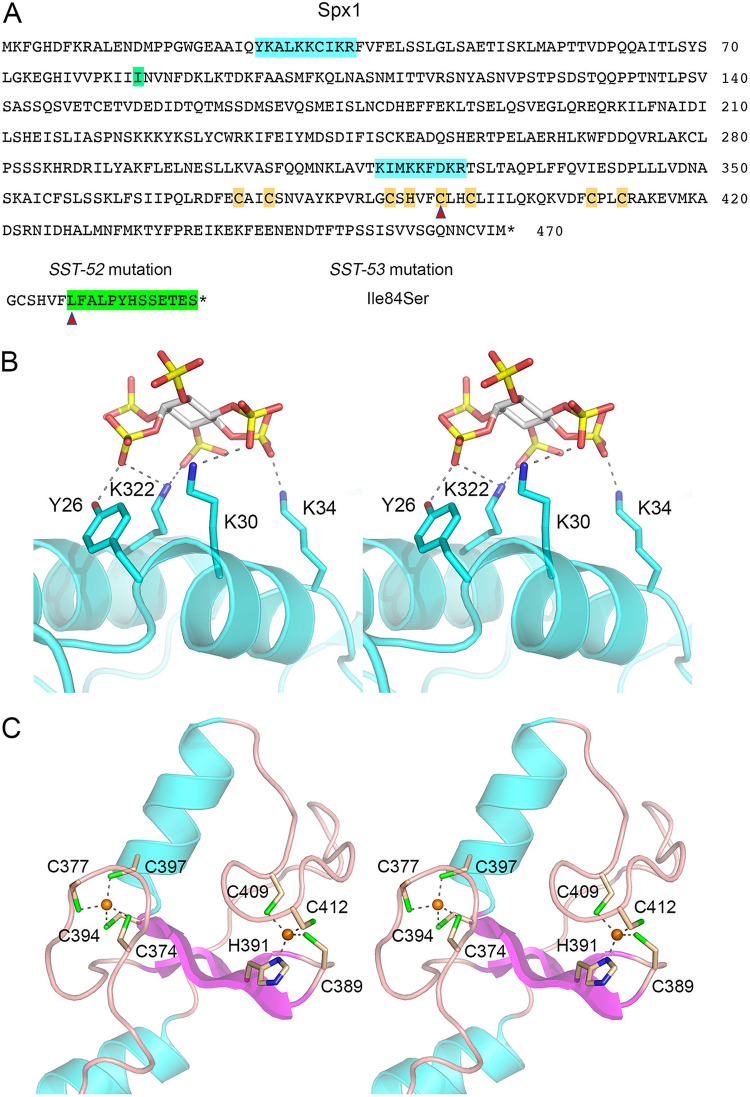
S. pombe Spx1. (A) Amino acid sequence of Spx1. The IPP-binding motifs in the SPX domain are highlighted in cyan. The zinc-binding residues of the ring finger domain are shaded in gold. The coding changes in the *SST-52* and *SST-53* alleles are shown at the bottom. (B) Structural model of the IPP-binding SPX domain of Spx1. Stereo view of a Phyre2 model of the IPP-binding site of Spx1, templated on the structure of Vtc4 bound to IP6 (PDB ID 5IJP). IP6 is depicted as a stick model with gray carbons and yellow phosphorus atoms. IP6-binding amino acid side chains of Spx1 are shown as stick models with cyan carbons. Atomic contacts to the IP6 phosphates are denoted by dashed lines. (C) Structural model of the Zn-binding RING domain of Spx1. Stereo view of a Phyre2 tertiary structure model of the ring finger domain of Spx1 (colored by secondary structure) templated on the crystal structure of the human E3 ligase NIRF (PDB ID 1Z6U). The two zinc atoms (gold spheres) are imported from the NIRF structure. The zinc-binding cysteine and histidine side chains of Spx1 are depicted as stick models with beige carbons.

The *SPAC6B12.07c* gene, which is inessential for fission yeast vegetative growth, encodes a 470-aa polypeptide (Fig. 3A) with a predicted N-terminal SPX domain (Fig. 3B) and a C-terminal zinc-binding RING finger domain with a characteristic C_3_HC_4_ motif spanning Cys374 to Cys412 (Fig. 3C). RING finger domains are found in ubiquitin E3 ligases (28). The putative IPP-binding motifs of the SPX domain are highlighted in cyan in Fig. 3A. Here, we refer to this fission yeast gene and protein as *spx1* and Spx1, respectively. The *SST-53* mutation in the *spx1* gene is a missense change in which Ile84 is replaced by serine (Fig. 3A). The *SST-52* allele is a +1 frameshift within the RING finger coding sequence that replaces the native polypeptide following Phe393 with a foreign 13-aa peptide, after which the mutant protein terminates at a new in-frame stop codon (Fig. 3A). The *SST-52* change results in deletion of three of the putative zinc-binding residues and thereby inactivation of the predicted E3 ligase.

### *spx1*Δ and *gde1*Δ suppress IPP toxicity.

**FIG 4:**
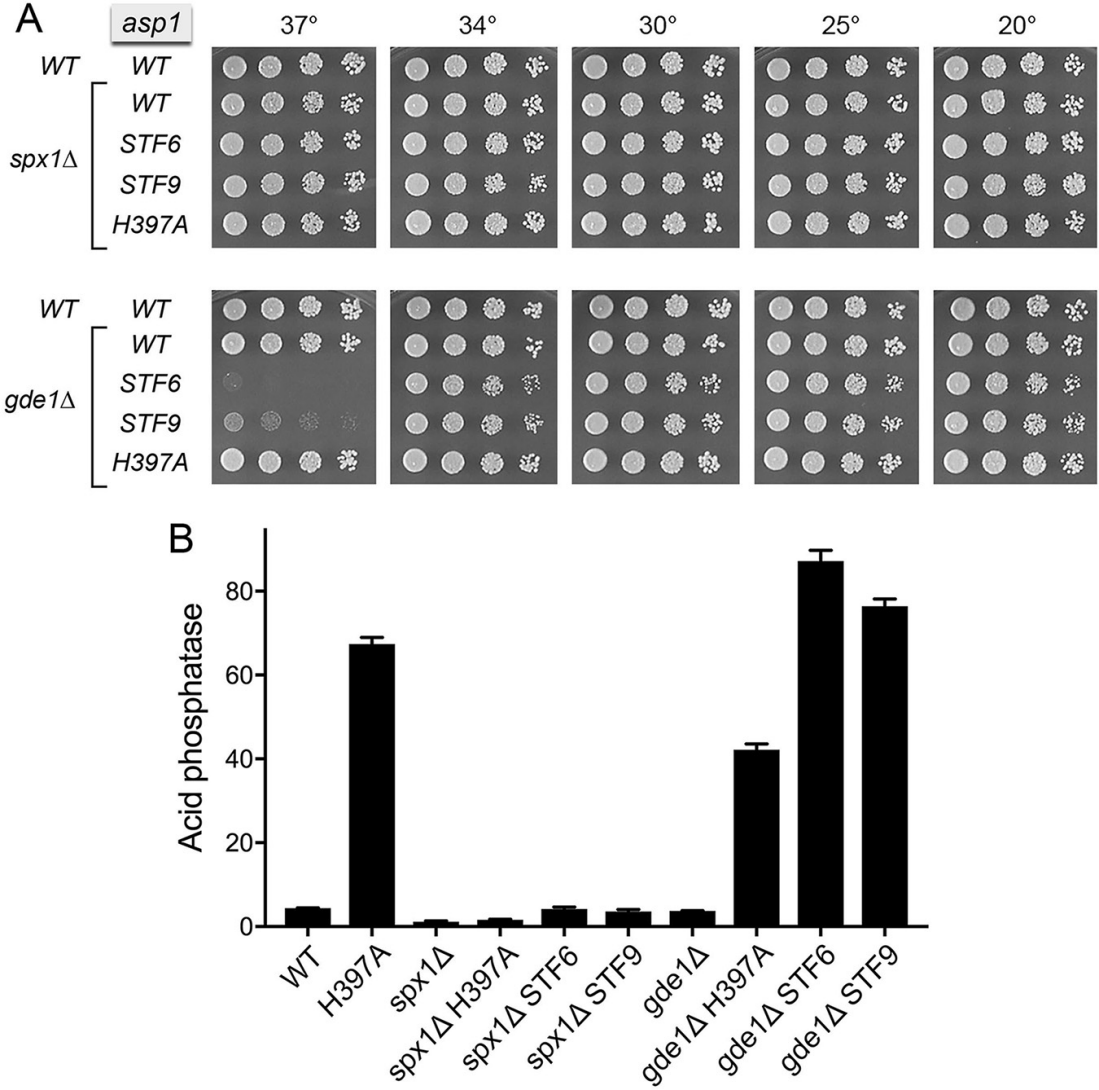
*spx1*Δ and *gde1*Δ suppress IPP toxicity. (A) Fission yeast strains with the indicated *asp1*, *spx1*, and *gde1* alleles were spot tested for growth on YES agar at the indicated temperatures. (B) The indicated strains were assayed for Pho1 acid phosphatase activity.

We constructed *spx1*Δ and *gde1*Δ strains in which the chromosomal *spx1*^+^ and *gde1*^+^ loci were deleted and replaced by drug-resistance markers. *spx1*Δ and *gde1*Δ cells grew as well as wild-type cells on YES agar at all temperatures tested (Fig. 4A). Whereas *spx1*Δ elicited a 4-fold hyperrepression of Pho1 expression in phosphate-replete cells compared to the wild-type control, Pho1 activity in *gde1*Δ cells was only 15% lower than in wild-type cells (Fig. 4B). To gauge whether a complete absence of these SPX proteins had the same capacity to suppress the effects of IPP pyrophosphatase mutations seen in the *STF* strains, we mated *spx1*Δ and *gde1*Δ to the *asp1-STF6 CTD-T4A* and *asp1-STF9 CTD-T4A* mutants. We also mated them to a viable *asp1-H397A* strain. After sporulation and screening of random spore populations for markers linked to the loci of interest, we obtained viable double-mutant haploid progeny: *spx1*Δ *asp1-H397A*, *spx1*Δ *STF6*, *spx1*Δ *STF9*, *gde1*Δ *asp1-H397A*, *gde1*Δ *STF6*, and *gde1*Δ *STF9* strains. The *spx1*Δ *asp1-H397A*, *spx1*Δ *STF6*, and *spx1*Δ *STF9* mutants grew as well as wild-type cells on YES agar at all temperatures tested (Fig. 4A), signifying that Spx1 is required to manifest the lethal effects of *STF6* and *STF9*. The strong derepression of Pho1 expression elicited by the *asp1-H397A* allele was effaced by *spx1*Δ (Fig. 4B). Pho1 expression was also hyperrepressed by *spx1*Δ in the double mutants bearing the *asp1 STF6* and *STF9* pyrophosphatase-defective alleles. We surmise that Spx1 is required to manifest the IP8-dependent precocious lncRNA 3′-processing/termination that underlies Pho1 derepression in phosphate-replete cells.

Whereas *gde1*Δ *asp1-H397A* cells grew like the wild-type, the *gde1*Δ *STF6* and *gde1*Δ *STF9* strains displayed a tight *ts* growth defect at 37°C (Fig. 4A), signifying that not all of the deleterious effects of the *STF6* and *STF9* alleles were eliminated in the absence of Gde1. These findings resonate with the modest *ts* phenotype observed in Fig. 1 for the Gde1 C-terminal truncation allele *SST-31* in the *STF3* background (*STF3* being less cytotoxic than either *STF6* or *STF9*). The impact of *gde1*Δ on Pho1 expression in the Asp1 pyrophosphatase-defective background was quite different from that seen for *spx1*Δ; to wit, (i) *gde1*Δ only moderately reduced (by 40%) the Pho1 derepression caused by *asp1-H397A* and (ii) Pho1 expression in *gde1*Δ *STF6* and *gde1*Δ *STF9* cells was derepressed to an even greater degree than in *asp1-H397A* (Fig. 4B). It would appear that *spx1*Δ is a “stronger” suppressor than *gde1*Δ, with respect to rescue of IPP toxicosis and reversal of IP8-dependent derepression of *pho1* expression.

We proceeded to construct a *gde1*Δ *spx1*Δ double-mutant strain that grew well on YES agar at 20 to 37°C (Fig. S2) and phenocopied the *spx1*Δ single mutant with respect to hyperrepression of Pho1 expression under phosphate-replete conditions (data not shown).

10.1128/mbio.03476-21.2 FIG S2 Growth of a *spx1*Δ *gde1*Δ double mutant. Serial 5-fold dilutions of wild-type and *spx1*Δ *gde1*Δ cells were spot tested for growth on YES agar at the indicated temperatures. Download FIG S2, TIF file, 0.7 MB. Copyright © 2022 Schwer et al. 2022 Schwer et al. https://creativecommons.org/licenses/by/4.0/ This content is distributed under the terms of the Creative Commons Attribution 4.0 International license.

### Effect of Gde1 active-site mutations and SPX domain mutations on Gde1 function in transducing IPP toxicity.

The *gde1 SST-31* mutation identified in the suppressor screen results in loss of the phosphodiesterase catalytic domain. A pertinent issue is whether the catalytic activity of Gde1 is required to transduce the toxicity of IPP pyrophosphatase mutations. Structures of glycerophosphodiesterase enzymes in complexes with metal cofactor and the glycerol-3-PO_4_ (G3P) reaction product reveal a conserved active site (26), depicted in Fig. S3A with amino acids numbered according to their position in fission yeast Gde1. The octahedral divalent cation coordination complex is occupied by the G3P O1 and O2 atoms, a G3P phosphate oxygen, Glu845-Oε, Asp847-Oδ, Glu968-Oε, and a water bridged to Glu845, Asp847, and Arg811. The three G3P phosphate oxygens are engaged to His810, Arg811, His860, and Lys970. Catalysis is thought to be a two-step chemical reaction entailing (i) attack by the glycerol O2 atom on the phosphorus of the phosphodiester, leading to expulsion of the R-OH product and formation of a glycerol-2,3-cyclic-phosphate intermediate, and (ii) attack by water on the cyclized phosphorus to generate G3P (26). The divalent cation and the basic amino acids that contact the phosphate presumably stabilize the transition state. The two histidines in the active site, which respectively coordinate the glycerol O2 and the bridging phosphate oxygen to the R-OH leaving group, are proposed to serve as general acid-base catalysts.

10.1128/mbio.03476-21.3 FIG S3 Gde1 active site mutations and SPX domain mutations. (A) Stereo view of the active site of a glycerophosphodiesterase enzyme (PDB ID 5T9C) in complex with metal cofactor (green sphere) and the glycerol-3-PO_4_ (G3P) reaction product, with amino acids shown as stick models and numbered according to their position in Gde1. (B) Stereo view of a Phyre2 model of the IPP-binding site of Gde1, templated on the crystal structure of Vtc4 bound to IP6 (PDB ID 5IJP). IP6 and IP6-binding amino acids side chains are shown as stick models. (C) Serial dilutions of fission yeast strains with the indicated *gde1* alleles were spot tested for growth on YES agar at the temperatures specified. Download FIG S3, JPG file, 0.5 MB. Copyright © 2022 Schwer et al. 2022 Schwer et al. https://creativecommons.org/licenses/by/4.0/ This content is distributed under the terms of the Creative Commons Attribution 4.0 International license.

**FIG 5:**
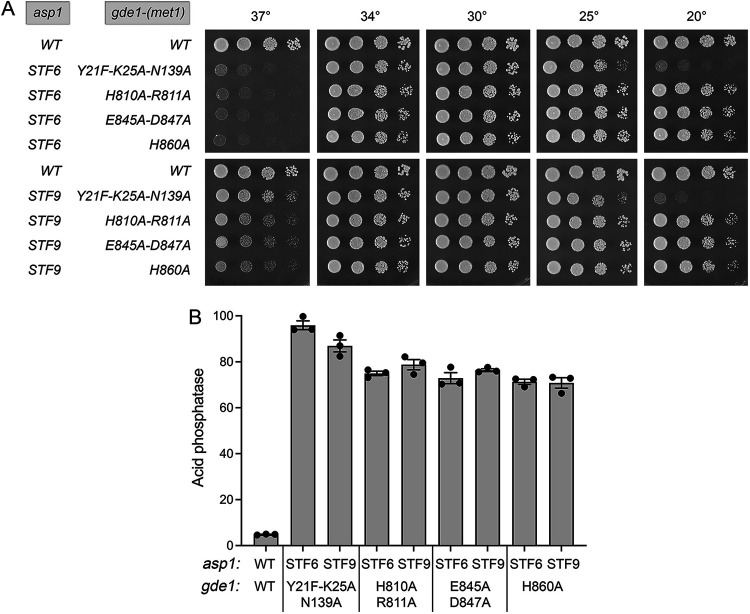
Effect of Gde1 active site and SPX mutations on Gde1 function in transducing IPP toxicity. (A) S. pombe strains bearing the indicated *asp1* and *gde1-*(*Met1*) alleles were spot tested for growth on YES agar at the indicated temperatures. (B) The indicated strains were assayed for Pho1 acid phosphatase activity.

Here, we replaced the chromosomal *gde1* gene with alleles encoding active-site alanine mutants H810A-R811A, E845A-D847A, and H860A; in each case we also replaced the Leu1 codon with a Met codon to install a canonical translation initiation site in the ORF. An isogenic strain encoding wild-type Gde1 with a Met1 codon was constructed to serve as a control. The *gde1-*(*met1*)*-WT* and three *gde1-*(*met1*)*-ala* strains, which grew well on YES agar at all temperatures (Fig. S3C), were crossed to *STF6 CTD-T4A* and *STF9 CTD-T4A* mutants to test for sensitivity or resistance to IPP toxicity. We recovered no viable *gde1-*(*met1*)*-WT STF6*, or *gde1-*(*met1*)*-WT STF9* haploid progeny, signifying that the Met1-initiated Gde1 was wild-type with respect to its conferral of sensitivity to IPP toxicity. In contrast, the *H810A-R811A*, *E845A-D847A*, and *H860A* alleles were all permissive for the recovery of viable *gde1-*(*met1*)*-ala STF6* and *gde1-*(*met1*)*-ala STF9* haploids that grew on YES agar at 20°C to 34°C but displayed a *ts* growth defect at 37°C (Fig. 5A), similar to the *gde1*Δ *STF6* and *gde1*Δ *STF9* strains (Fig. 4A). We surmise that the glycerophosphodiesterase catalytic activity of Gde1 is required for IPP toxicity inflicted by Asp1 pyrophosphatase mutations. Pho1 acid phosphatase expression in *gde1-H810A-R811A STF6*, *gde1-H810A-R811A STF9*, *gde1-E845A-D847A STF6*, *gde1-E845A-D847A STF9*, *gde1-H860A STF6*, and *gde1-H860A STF9* cells was derepressed by 15- to 16-fold versus wild-type cells (Fig. 5B).

Phyre2 generated a tertiary structure model of the Gde1 SPX domain that we superimposed on the structure of the SPX domain of Chaetomium thermophilum Vtc4 in complex with IP6 (4). The IP6 ligand from that superimposed structure (PDB ID 5IJP) is shown in complex with the Gde1 Phyre2 model in Fig. S3B. A Tyr22-Lys26-Lys147 triad that binds the IP6 phosphates in the Vtc4 structure corresponds to Tyr21-Lys25-Asn139 in the fission yeast Gde1 model, where Tyr21 and Lys25 are within hydrogen bonding distance of IP6 phosphates and Asn139 Nδ is within van der Waals distance of an IP6 phosphate (Fig. S3B). To test the effect of perturbing the putative IPP binding surface of Gde1, we introduced a triple mutation, *Y21F-K25A-N139A*, into the chromosomal *gde1* gene along with a Met1 codon. After crossing the *gde1-*(*met1*)*-Y21F-K25A-N139A* cells, which grew well on YES agar at all temperatures (Fig. S3C), to *STF6 CTD-T4A* and *STF9 CTD-T4A* cells, we recovered viable *gde1-*(*met1*)*-Y21F-K25A-N139A STF6* and *gde1-*(*met1*)*-Y21F-K25A-N139A STF9* haploids that grew on YES agar at 30°C and 34°C but displayed both *ts* and *cs* growth defects at 37°C and 20°C, respectively (Fig. 5A). Whereas we infer from these results that IPP binding by the SPX domain of Gde1 is important for IPP toxicity in the context of Asp1 pyrophosphatase mutations, it is evident that Gde1 retains some function in this regard at colder temperatures when the IPP-binding site is mutated. This hints that the catalytic activity of Gde1 might not be strictly dependent on SPX-IPP interaction. Pho1 expression in *gde1-Y21F-K25A-N139A STF6* and *gde1-Y21F-K25A-N139A STF9* cells was derepressed by 20-fold and 18-fold relative to wild-type cells (Fig. 5B).

### *spx1*Δ, but not *gde1*Δ, suppresses the synthetic lethality of *aps1*Δ *asp1-H397A*.

**FIG 6:**
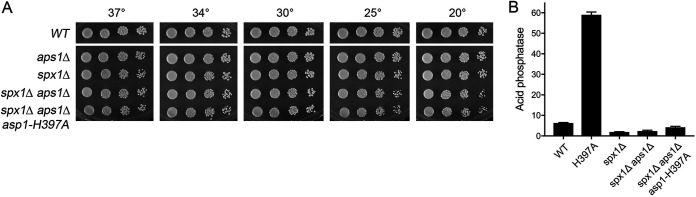
*spx1*Δ, but not *gde1*Δ, suppresses the synthetic lethality of *aps1*Δ *asp1-H397A*. (A) Fission yeast cells with genotypes as specified on the left were spot tested for growth on YES agar at the indicated temperatures. (B) The indicated strains were assayed for Pho1 acid phosphatase activity.

The initial finding that simultaneous inactivation of two IPP pyrophosphatase enzymes (by deletion of Aps1 and pyrophosphatase active site mutation of Asp1) was lethal ushered in our concept of IPP toxicosis via unconstrained precocious termination (12). Thus, it was of interest to test if the *spx1*Δ and *gde1*Δ mutations that suppress lethality of *asp1-STF* single mutants might also rescue the synthetic lethality of *aps1*Δ *asp1-H397A*. To do this, we mated *spx1*Δ *asp1-H397A* cells with *aps1*Δ cells and *gde1*Δ *asp1-H397A* cells with *aps1*Δ cells, sporulated the resulting diploids, and screened random spores for each of the differentially marked loci of interest. In this way, we recovered viable *spx1*Δ *aps1Δ asp1-H397A* haploid progeny. *spx1*Δ *asp1-H397A aps1Δ* cells grew as well as the parental *spx1*Δ *aps1*Δ strain at all temperatures tested (Fig. 6A). In contrast, we recovered no viable *gde1*Δ *aps1Δ asp1-H397A* haploids. We surmise that *aps1Δ asp1-H397A* exerts stronger IP8-driven toxicity than *asp1-STF6* or *asp1-STF9*. Whereas *spx1*Δ suppresses this extreme toxicity, *gde1*Δ cannot.

Previous studies showed that simultaneous inactivation of the Asp1 and Aps1 pyrophosphatases additively derepressed Pho1 expression (to acid phosphatase activity levels of >150) in triple mutants in which the synthetic lethality of *aps1Δ asp1-H397A* had been rescued by any of several CPF mutations (12). We find here that *spx1*Δ erased *pho1* derepression by *aps1Δ asp1-H397A* (Fig. 6B).

### *spx1*Δ interdicts Pho1 derepression by Pol2 CTD mutations.

**FIG 7:**
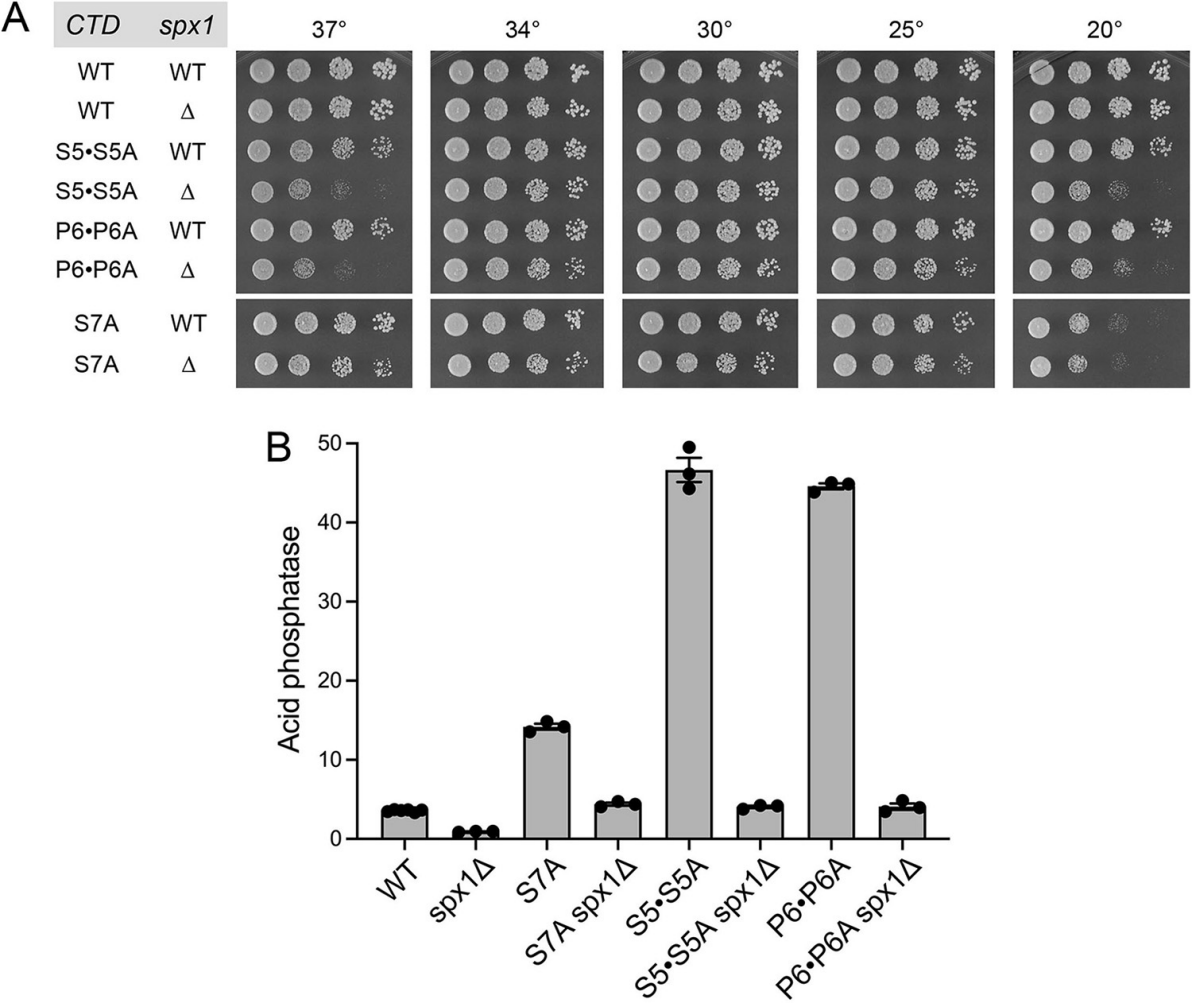
*spx1*Δ interdicts Pho1 derepression by Pol2 CTD mutations. (A) Serial 5-fold dilutions of fission yeast strains (as specified on the left) were spot tested for growth on YES agar at the indicated temperatures. (B) The indicated strains were assayed for Pho1 acid phosphatase activity.

The carboxy-terminal domain (CTD) of the Rpb1 subunit of fission yeast Pol2 consists of 29 tandem repeats of the consensus heptapeptide Y^1^S^2^P^3^T^4^S^5^P^6^S^7^. Genetic perturbations of the Pol2 CTD can either derepress or hyperrepress Pho1 expression in phosphate-replete cells by virtue of enhancing or reducing the propensity to cleave and polyadenylate nascent *prt* lncRNA and terminate *prt* lncRNA transcription prior to reaching the flanking *pho1* mRNA promoter. For example, derepression occurs when (i) every Ser7 residue is mutated to alanine in *CTD-S7A* cells, (ii) Ser5 is changed to alanine in every other heptad in *CTD-S5·S5A* cells, and (iii) Pro6 is changed to alanine in every other heptad in *CTD-P6·P6A* cells (10, 18, 29) (Fig. 7B). In light of the finding that Spx1 is strictly required for Pho1 derepression in cells lacking Asp1 and Aps1 IPP pyrophosphatase activity (Fig. 6B), we sought to gauge if the derepressive effect of CTD alleles *S5·S5A*, *P6·P6A*, and *S7A* on *pho1* depends similarly on Spx1. We mated *S5·S5A*, *P6·P6A*, and *S7A* strains with a *spx1*Δ strain and recovered viable *S5·S5A spx1*Δ, *P6·P6A spx1*Δ, and *S7A spx1*Δ double mutants that grew as well as the respective parental CTD mutants on YES agar at 30°C (Fig. 7A). Introducing *spx1*Δ into the *S5·S5A* and *P6·P6A* backgrounds elicited *ts* and *cs* growth phenotypes at 37°C and 20°C, respectively (Fig. 7A). The salient findings were that the Pho1 derepression in *S5·S5A* (13-fold), *P6·P6A* (12-fold), and *S7A* (4-fold) cells vis-à-vis the wild-type was eliminated in the absence of Spx1, such that Pho1 activity in the double mutants was reduced to the level seen in wild-type cells (Fig. 7B).

### *spx1*Δ squelches Pho1 derepression elicited by a gain-of-function mutation in termination factor Seb1.

**FIG 8:**
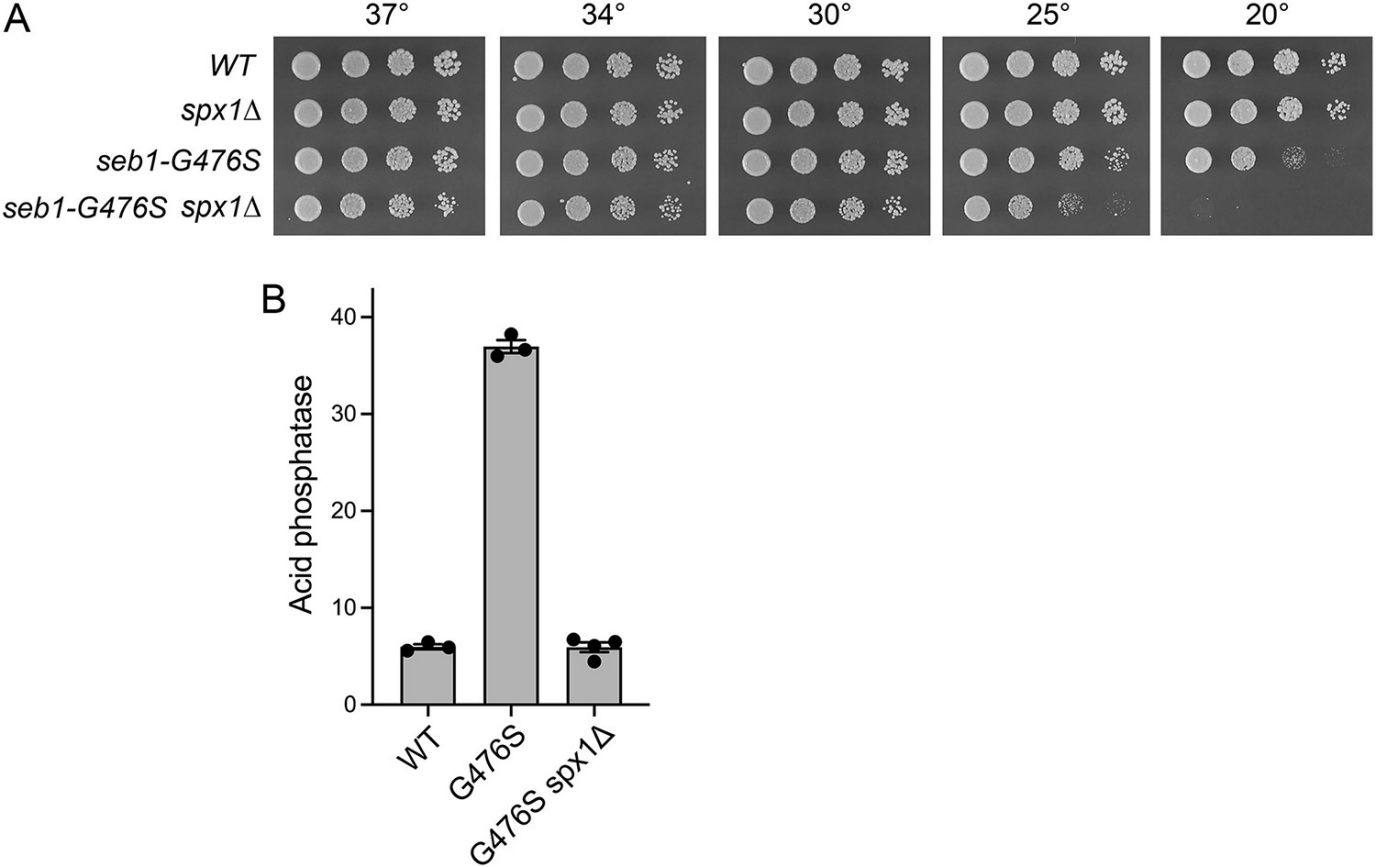
*spx1*Δ squelches Pho1 derepression by a gain-of-function mutation in termination factor Seb1. (A) Serial 5-fold dilutions of fission yeast strains (as specified on the left) were spot tested for growth on YES agar at the indicated temperatures. (B) The indicated strains were assayed for Pho1 acid phosphatase activity.

By performing a forward genetic screen for relief of lncRNA interference with *pho1* expression, we uncovered a mutation, G476S, in the RNA-binding domain of essential termination factor Seb1 that evokes precocious lncRNA termination in response to 5′-proximal poly(A) sites in a manner dependent on the cleavage and polyadenylation factor (CPF), the termination factor Rhn1, and inositol pyrophosphate synthesis (30). Multiple lines of evidence point to Seb1-G476S as a unique gain-of-function mutation in a Pol2 transcription termination factor (30). To query epistasis between *seb1-G476S* and *spx1*Δ, we constructed a *seb1-G476S spx1*Δ double mutant, which grew well on YES agar at 30°C to 37°C (Fig. 8A). *spx1*Δ exacerbated the cold-sensitive phenotype of the *seb1-G476S* single-mutant (slow growth at 20°C) such that *seb1-G476S spx1*Δ cells failed to grow at 20°C and grew slowly at 25°C (Fig. 8A). We found that the Pho1 derepression in *seb1-G476S* cells *vis-à-vis* wild-type was attenuated in the absence of Spx1, such that Pho1 activity in the double mutant was reduced to the level seen in wild-type cells (Fig. 8B).

### Transcriptome profiling of the *spx1Δ* strain.

We performed transcriptome sequencing (RNA-seq) on poly(A)^+^ RNA isolated from *spx1Δ* cells and from the parental wild-type strain. cDNAs obtained from three biological replicates (using RNA from cells grown to mid-log phase in YES medium at 30°C) were sequenced for each strain. Read densities for individual genes were highly reproducible between biological replicates (Pearson coefficients of 0.95 to 0.99). A cutoff of ±2-fold change in normalized transcript read level and an adjusted *P* value of ≤0.05 were the criteria applied to derive an initial list of differentially expressed annotated loci in the *spx1Δ* mutant versus the parental wild-type control. We then focused on differentially expressed genes with average normalized read counts of ≥100 in either strain in order to eliminate transcripts that were expressed at very low levels in vegetative cells. We thereby identified 173 annotated protein-coding genes that were upregulated by these criteria in *spx1Δ* cells and 132 coding genes that were downregulated (Table S1).

10.1128/mbio.03476-21.8 TABLE S1 Summary of RNA-seq analysis of the *spx1*Δ strain versus the wild-type. Lists of coding and noncoding RNAs that were dysregulated (UP or DOWN) by ≥2-fold. Download Table S1, XLSX file, 0.1 MB. Copyright © 2022 Schwer et al. 2022 Schwer et al. https://creativecommons.org/licenses/by/4.0/ This content is distributed under the terms of the Creative Commons Attribution 4.0 International license.

**FIG 9:**
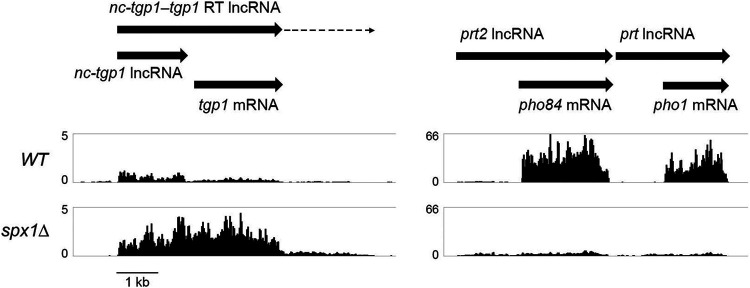
*spx1*Δ dysregulates transcription of the fission yeast *PHO* regulon. Strand-specific RNA-seq read densities (counts/base/million, averaged in a 25-nucleotide [nt] window) of the indicated strains are plotted on the *y* axis as a function of position across the *nc-tgp1–tgp1*, *prt2–pho84*, and *prt–pho1* loci (*x* axis). The read densities were determined from cumulative counts of all three RNA-seq replicates for each S. pombe strain. The *y* axis scale for each track is indicated. The common *x* axis scale is shown on the bottom left. The individual lncRNA or mRNA transcripts are labeled and shown to scale as black arrows in the direction of their synthesis. The broken arrow indicates an extended *nc-tgp1–tgp1* read-through lncRNA.

The most highly downregulated subset (≥8-fold decrement) embraced seven genes, including phosphate homeostasis genes *pho1* and *pho84*. Inspection of the RNA-seq reads at the tandem chromosomal *pho84 pho1* gene cluster (Fig. 9) showed that (i) the *pho84* and *pho1* mRNAs that were present in wild-type cells were eliminated in *spx1*Δ cells and (ii) the residual transcripts from these two loci present in *spx1*Δ cells corresponded to the *prt2* and *prt* lncRNAs that initiate from upstream lncRNA promoters and terminate at the *pho84* and *pho1* poly(A) sites, respectively. Moreover, the read counts over the *prt2* and *prt* lncRNA transcription units preceding the mRNA start site are higher (by 2.4-fold and 3.4-fold, respectively) in *spx1*Δ cells than wild-type cells, consistent with increased lncRNA transcriptional interference as the basis for the Pho1 hyperrepression seen in *spx1*Δ cells. The subset of 23 genes that were upregulated by ≥8-fold included 11 genes connected with fission yeast meiosis. We were initially surprised to find that the *PHO* gene *tgp1* was upregulated 8-fold in *spx1*Δ cells, this being a rare case in which there is such a divergent effect on expression levels within the *PHO* regulon. However, inspection of the reads across the *tgp1* locus showed clearly that the apparent increase in RNAs derived from the *tgp1* ORF was caused by an increase in the production of a *nc-tgp1–tgp1* read-through lncRNA in *spx1*Δ cells (evinced by a 3.4-fold increase in read density over the *nc-tgp1* lncRNA segment preceding the *tgp1* transcript) rather than an increase in the *tgp1* mRNA (Fig. 9).

### Effect of SPX domain mutations and RING finger mutations on Spx1 function in transducing IPP toxicity.

**FIG 10:**
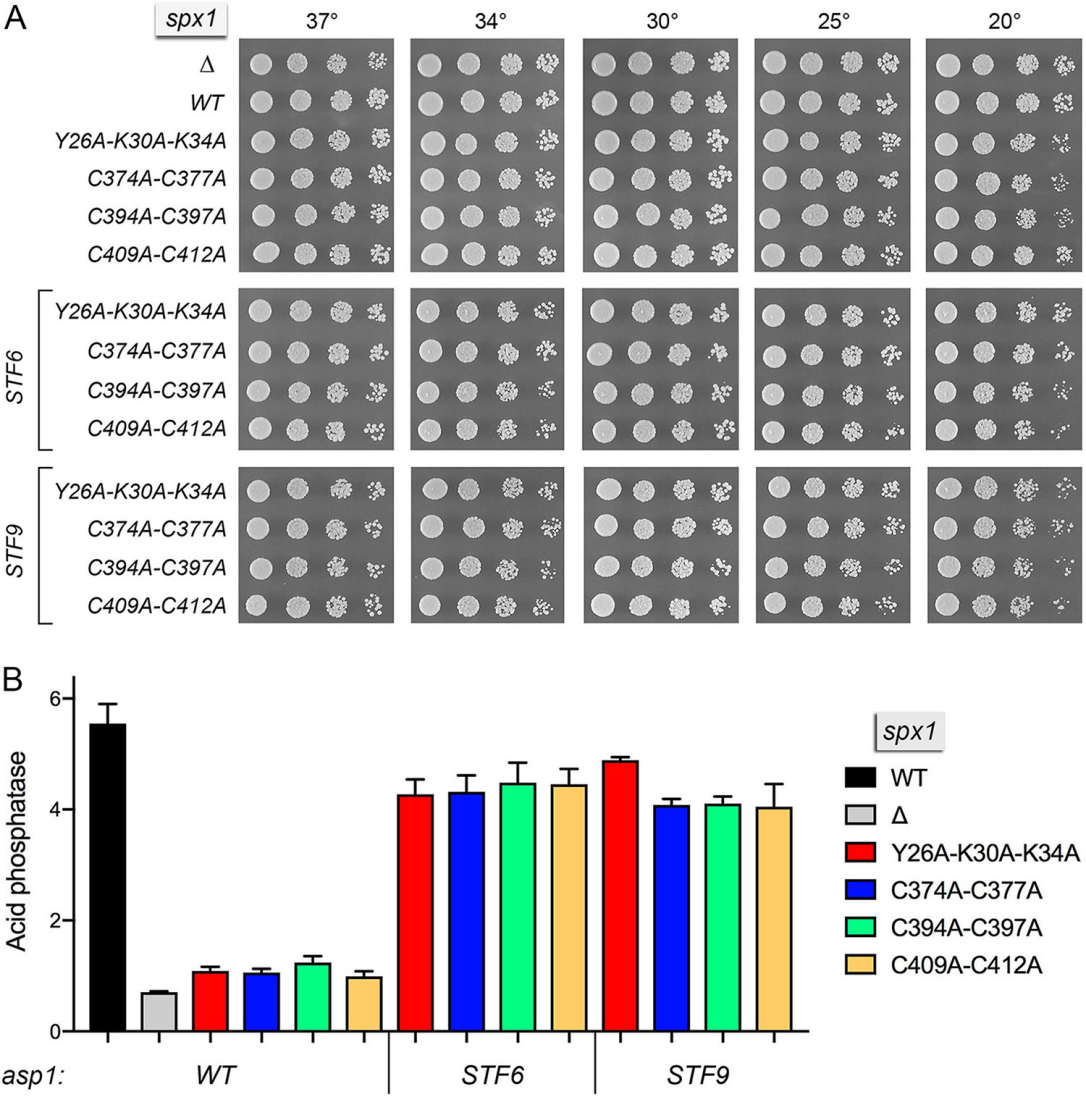
Effect of SPX and RING finger mutations on Spx1 function in transducing IPP toxicity. (A) Serial 5-fold dilutions of fission yeast strains bearing the indicated *spx1* alleles in *asp1*-*WT*, -*STF6*, or -*STF9* backgrounds were spot tested for growth on YES agar at the indicated temperatures. (B) The indicated strains were assayed for Pho1 acid phosphatase activity.

The SPX domain of Spx1 spans aa 1 to 350. A Phyre2 tertiary structure model of the IPP-binding site of Spx1, templated on the Vtc4 crystal structure (4), is shown in Fig. 3B. The N-terminal IPP-binding ^22^YxxxKxxxK^30^ motif of Vtc4 is conserved in Spx1 as ^26^YxxxKxxxK^34^. To interrogate the requirement for the Spx1 IPP-binding site, we introduced a *Y26A-K30A-K34A* triple-alanine mutant allele at the chromosomal *spx1* locus, flanked by a G418 resistance marker. The *spx1-Y26A-K30A-K34A* strain grew as well as the wild-type on YES agar (Fig. 10A). Phyre2 predicted a tertiary structure for the RING finger domain of Spx1 (aa 336 to 438) (Fig. 3C) templated on the crystal structure of the human E3 ligase NIRF (PDB ID 1Z6U). Alignment of these two RING domains highlighted 33 positions of amino acid side chain identity/similarity within the homologous 103-aa polypeptide segment. To probe whether the putative E3 ligase activity of Spx1 is required to transduce the toxicity of IPP pyrophosphatase mutations, we made a series of three double-alanine mutations of the zinc-binding RING finger amino acids (shown in Fig. 3C, in which the two zinc atoms from PDB entry 1Z6U were superimposed on the Phyre2 model of Spx1). The *spx1* alleles *C374A-C377A*, *C394A-C397A*, and *C409A-C412A* were introduced at the chromosomal *spx1* locus. These *spx1-*(*CC-AA*) strains grew as well as the wild-type on YES agar (Fig. 10A).

To see if the IPP-binding site and RING finger mutations conferred resistance to IPP toxicity, the *Y26A-K30A-K34A*, *C374A-C377A*, *C394A-C397A*, and *C409A-C412A* strains were crossed to *STF6 CTD-T4A* and *STF9 CTD-T4A* mutants. We selected serially a large population of random spores for the drug-resistance markers flanking the *asp1 STF6* and *STF9* alleles and for the resistance gene flanking *spx1* and then screened for absence of the nourseothricin-resistance marker linked to *rpb1-CTD-T4A*. We thereby recovered viable *spx1-Y26A-K30A-K34A asp1-STF6* and *spx1-Y26A-K30A-K34A asp1-STF9* cells and all six combinations of viable *spx1-*(*CC-AA*) *asp1-STF6* and *spx1-*(*CC-AA*) *asp1-STF9* haploids that grew on YES agar at all temperatures tested (Fig. 10A), thus phenocopying *spx1*Δ in this regard. We surmise that IPP binding by Spx1 and the integrity of the Spx1 RING finger are necessary for *asp1-STF* mutants to elicit lethal IPP toxicosis. The *spx1-Y26A-K30A-K34A*, *C374A-C377A*, *C394A-C397A*, and *C409A-C412A* alleles also mimicked *spx1*Δ in hyperrepressing Pho1 and blocking derepression of Pho1 in the *STF6* and *STF9* backgrounds (Fig. 10B), signifying that IPP-binding and the RING finger are both required to transduce IPP signaling that alleviates transcriptional interference at the *prt–pho1* locus.

### Deletions of SPX domain-containing Vtc2 and Vtc4 subunits of vacuolar polyphosphate polymerase suppress lethal IPP toxicity.

The fission yeast proteome includes four other SPX domain proteins: Vtc4, Vtc2, Plt1, and SPCC1827.07c. Vtc4 and Vtc2 are paralogous subunits of the yeast vacuolar transporter chaperone (VTC) complex that synthesizes inorganic polyphosphate and simultaneously imports the polyphosphate into the yeast vacuole (21, 31). Polyphosphate synthesis by the VTC requires the proton gradient across the vacuolar membrane established by the resident V-type H^+^-ATPase (31). Vtc4 is the catalytic subunit of the polyphosphate polymerase; it consists of (i) a cytoplasmically facing N-terminal SPX domain and central polymerase domain and (ii) a C-terminal membrane anchor domain. The Vtc4 polymerase domain, which catalyzes manganese-dependent transfer of a nucleoside triphosphate (NTP) γ-phosphate to an inorganic pyrophosphate or phosphate primer (21), is a member of the triphosphate tunnel metalloenzyme (TTM) family (32, 33). Vtc2 is homologous to Vtc4, but its TTM domain is catalytically inactive. Polyphosphate synthesis by the budding yeast VTC complex in isolated vacuoles is stimulated ∼20-fold by submicromolar concentrations of IPPs, with 1,5-IP8 being at least 20-fold more potent than 5-IP7 or 1-IP7 based on the 50% effective concentration (EC_50_) (34). Simultaneous missense mutation of single amino acids in the IPP-binding sites of both SPX domains within the vacuolar VTC complex abolished IP7-stimulated polyphosphate synthesis (4).

**FIG 11:**
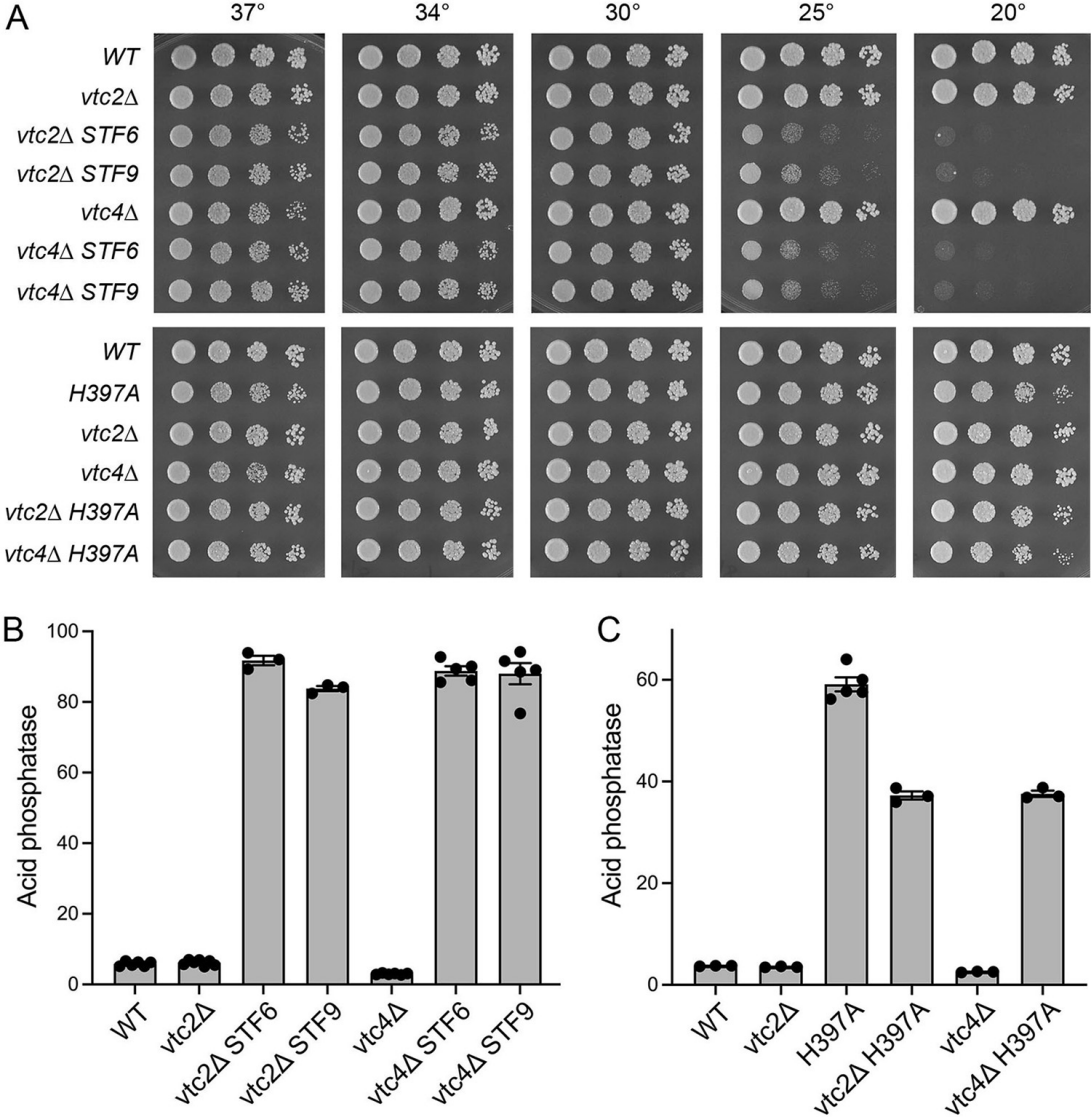
Deletions of SPX domain-containing Vtc2 and Vtc4 subunits of vacuolar polyphosphate polymerase suppress lethal IPP toxicity. (A) Serial 5-fold dilutions of fission yeast strains bearing the indicated *vtc2* or *vtc4* alleles in *asp1*-*WT*, -*STF6*, -*STF9*, or -*H397A* backgrounds were spot tested for growth on YES agar at the indicated temperatures. (B) The indicated strains were assayed for Pho1 acid phosphatase activity.

Here, we queried whether the SPX domain VTC subunits Vtc4 and Vtc2 might play a role in transducing toxicity signals generated in Asp1 pyrophosphatase mutant backgrounds. We constructed *vtc4*Δ and *vtc2*Δ strains in which the respective open reading frames were deleted and replaced by drug-resistance markers. *vtc2*Δ cells grew as well as wild-type cells on YES agar at all temperatures tested; *vtc4*Δ cells grew like wild-type cells at 20 to 34°C but formed slightly smaller colonies at 37°C (Fig. 11A). Pho1 acid phosphatase activity in *vtc2*Δ cells was identical to that in wild-type cells; Pho1 expression in *vtc4*Δ cells was half that in wild-type cells (Fig. 11B). After mating *vtc4*Δ and *vtc2*Δ to *asp1-STF6 CTD-T4A* and *asp1-STF9 CTD-T4A* strains and screening a random spore population for markers linked to the loci of interest, we obtained viable double-mutant *vtc4*Δ *STF6*, *vtc4*Δ *STF9*, *vtc2*Δ *STF6*, and *vtc2*Δ *STF9* haploid progeny, signifying that loss of SPX-domain VTC subunits suppressed lethal IPP toxicosis. The *vtc4*Δ *STF6*, *vtc4*Δ *STF9*, *vtc2*Δ *STF6*, and *vtc2*Δ *STF9* strains grew well at 30°C to 37°C but were sick at 25°C and failed to form colonies at 20°C (Fig. 11A). Pho1 activity in *vtc4*Δ *STF6*, *vtc4*Δ *STF9*, *vtc2*Δ *STF6*, and *vtc2*Δ *STF9* cells was derepressed by 15-fold versus that in wild-type cells (Fig. 11B). Thus, it appeared that *vtc2*Δ and *vtc4*Δ were less potent suppressors of IPP toxicity and less potent squelchers of IPP-dependent Pho1 derepression than *spx1*Δ.

To focus on the potential contributions of the VTC complex to IP8-driven upregulation of *pho1* in the absence of IPP toxicosis, we mated *vtc4*Δ and *vtc2*Δ strains to a *asp1-H397A* strain and compared Pho1 expression in the *vtc4*Δ *asp1-H397A* and *vtc2*Δ *asp1-H397A* double mutants to that in the *asp1-H397A* single mutant. The Vtc4 and Vtc2 deletions reduced Pho1 expression in *asp1-H397A* cells by 37% (Fig. 11C), which mimics the 40% reduction in *gde1*Δ *asp1-H397A* versus *asp1-H397A* cells but contrasts sharply with the 98% reduction seen in *spx1*Δ *asp1-H397A* versus *asp1-H397A* (Fig. 4B).

To test if *vtc4*Δ could suppress the synthetic lethality of *aps1*Δ *asp1-H397A*, we mated the *vtc4*Δ *asp1-H397A* strain with the *aps1Δ* strain, sporulated the resulting diploids, and screened a large population of random spores for each of the differentially marked loci of interest. No viable *vtc4*Δ *aps1Δ asp1-H397A* haploids were recovered.

### Effect of Vtc4 polyphosphate polymerase active site and SPX domain IPP binding site mutations on Vtc4 function in transducing IPP toxicity.

A pertinent issue is whether the catalytic activity of Vtc4 in polyphosphate synthesis is required to manifest the toxicity of IPP pyrophosphatase mutations. Structure-guided mutagenesis of the active site of the catalytic domain of budding yeast Vtc4 identified ATP-binding residues Arg264 and Arg266 as essential for polyphosphate synthesis *in vitro* and for cellular polyphosphate accumulation *in vivo* (21). The equivalent active site residues in fission yeast Vtc4 are Arg262 and Arg264. Here, we replaced the chromosomal *vtc4* gene with an allele encoding the active site R262A-R264A mutant and a downstream flanking G418 resistance marker. The *vtc4-R262A-R264A* strain grew as well as an isogenic *vtc4-WT* strain bearing the same flanking marker (Fig. S4).

10.1128/mbio.03476-21.4 FIG S4 Polyphosphate polymerase activity is required for Vtc4 function in transducing IPP toxicity. Serial 5-fold dilutions of cells with *vtc4* and *asp1* genotypes specified at left were spot tested for growth on YES agar at the indicated temperatures. Download FIG S4, TIF file, 1.2 MB. Copyright © 2022 Schwer et al. 2022 Schwer et al. https://creativecommons.org/licenses/by/4.0/ This content is distributed under the terms of the Creative Commons Attribution 4.0 International license.

Mating the *vtc4-R262A-R264A* strain to *STF6 CTD-T4A* and *STF9 CTD-T4A* mutants, followed by screening of large populations of random spores for the drug resistance markers flanking the *vtc4* and *asp1* alleles, yielded viable *vtc4-R262A-R264A asp1-STF6* and *vtc4-R262A-R264A asp1-STF9* cells that grew well on YES agar at 30 to 37°C but were slow growing at 25°C and failed to form macroscopic colonies at 20°C (Fig. S4), thereby phenocopying *vtc4*Δ in this regard. We conclude that the polyphosphate polymerase activity of Vtc4 is necessary for *asp1-STF* mutants to trigger lethal IPP toxicosis.

The SPX domain of fission yeast Vtc4 spans aa 1 to 350 and includes a conserved N-terminal IPP-binding ^22^YxxxKxxxK^30^ motif (4). To interrogate the requirement for the Vtc4 IPP-binding site, we introduced a *Y22A-K26A-K30A* triple-alanine mutant allele at the chromosomal *vtc4* locus, flanked by a G418 resistance marker. The *vtc4-Y22A-K26A-K30A* strain grew as well as the *vtc4-WT* strain on YES agar (Fig. S4).

After mating of the *vtc4-Y22A-K26A-K30A* strain to *STF6 CTD-T4A* and *STF9 CTD-T4A* mutants and ensuing random spore analysis, we were unable to obtain viable *vtc4-Y22A-K26A-K30A asp1-STF6* or *vtc4-Y22A-K26A-K30A asp1-STF9* haploid progeny, signifying that the IPP-binding site in the Vtc4 SPX domain is not necessary to elicit IPP toxicosis.

### Deletions of SPX proteins Plt1 and SPCC1827.07c (Spx2) do not suppress lethal IPP toxicity.

There are two other SPX domain-containing proteins in the fission yeast proteome. Plt1, a putative low-affinity transmembrane inorganic phosphate transporter homologous to budding yeast Pho87, is an 867-aa polypeptide composed of an N-terminal SPX domain and a C-terminal transporter domain. SPCC1827.07c, a putative membrane protein homologous to budding yeast Syg1, is a 682-aa protein composed of an N-terminal SPX domain and a C-terminal EXS domain. Here, we refer to SPCC1827.07c as Spx2. We constructed *plt1*Δ and *spx2*Δ strains in which the respective open reading frames were deleted and replaced by drug resistance markers. *spx2*Δ cells grew as well as wild-type cells on YES agar at all temperatures tested (Fig. S5A). *plt1*Δ cells grew like wild-type cells at 20°C to 34°C but formed smaller colonies at 37°C (Fig. S5A). Pho1 acid phosphatase activity in phosphate-replete *plt1*Δ and *spx2*Δ cells was 70% of wild-type activity (Fig. S5B). After mating *plt1*Δ and *spx2*Δ to *asp1-STF6 CTD-T4A* and *asp1-STF9 CTD-T4A* strains and screening a large random spore population for markers linked to the loci of interest, we did not recover any viable *plt1*Δ *STF6*, *plt1*Δ *STF9*, *spx2*Δ *STF6*, or *spx2*Δ *STF9* haploid progeny. Thus, it is not the case that every SPX protein plays a role in transducing IPP toxicity of *asp1-STF6* and *asp1-STF9* alleles.

10.1128/mbio.03476-21.5 FIG S5 Deletions of SPX proteins SPCC1827.07c (Spx2) and Plt1. (A) Serial 5-fold dilutions of wild-type, *spx2Δ*, and *plt1*Δ cells were spot tested for growth on YES agar at the indicated temperatures. (B) The indicated strains were assayed for Pho1 acid phosphatase activity. Download FIG S5, TIF file, 2.6 MB. Copyright © 2022 Schwer et al. 2022 Schwer et al. https://creativecommons.org/licenses/by/4.0/ This content is distributed under the terms of the Creative Commons Attribution 4.0 International license.

### *spx1*Δ delays the onset of the phosphate starvation response.

**FIG 12:**
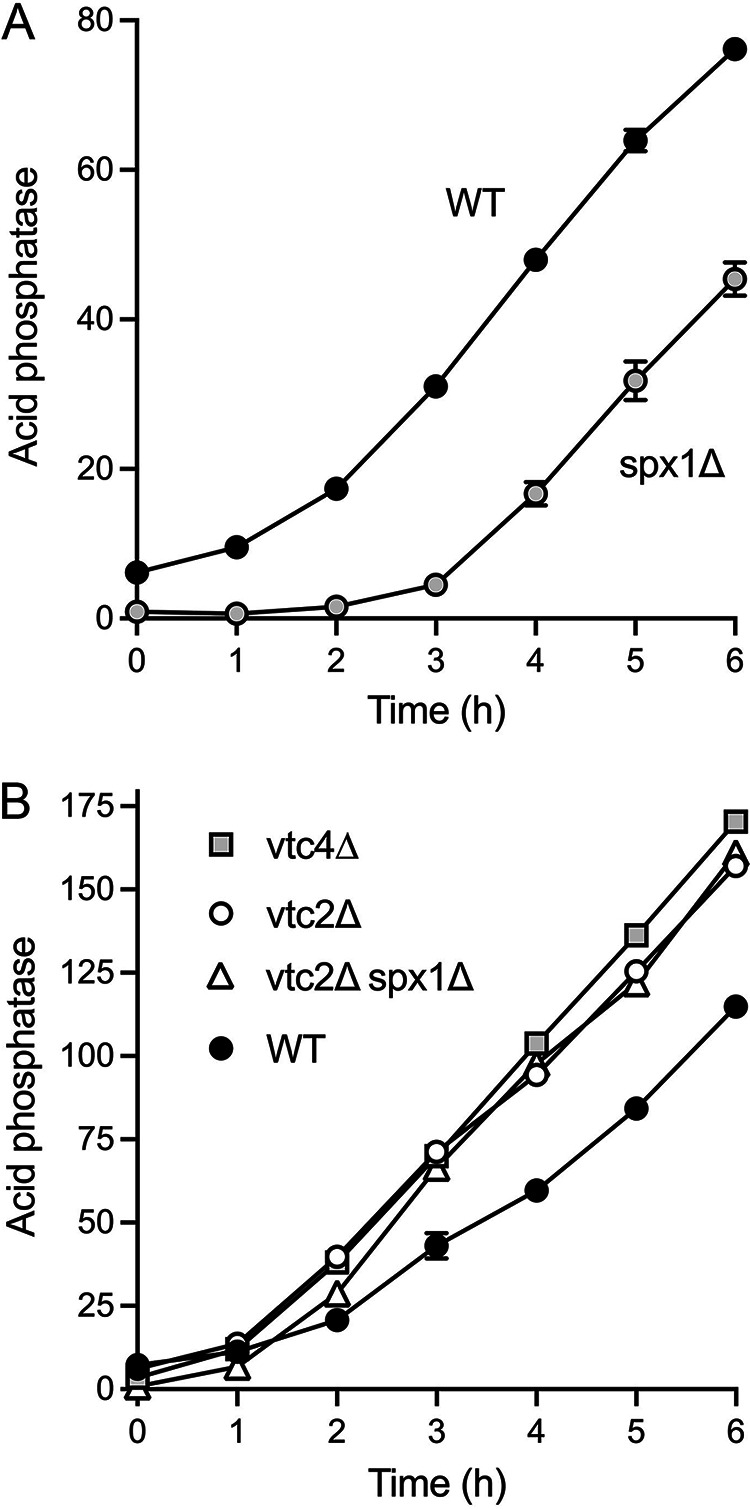
*spx1*Δ delays the onset of the phosphate starvation response. (A) Wild-type and *spx1*Δ cells were assayed for Pho1 acid phosphatase activity prior to (time zero) and at hourly intervals after transfer to PMG medium lacking phosphate. (B) Time course of Pho1 induction in response to phosphate starvation in *vtc4Δ*, *vtc2Δ*, and *vtc2Δ spx1Δ* cells. The indicated strains were assayed for Pho1 acid phosphatase activity prior to (time zero) and at hourly intervals after transfer to PMG medium lacking phosphate. Data are the averages and SEM from three independent experiments.

Wild-type and *spx1*Δ cells were grown in liquid culture in YES medium, washed with water, and then incubated in synthetic medium lacking exogenous phosphate. Aliquots of the cultures were assayed for Pho1 acid phosphatase activity prior to and at hourly intervals after transfer to phosphate-free medium. Wild-type cells respond to phosphate starvation by derepressing *pho1* transcription and thereby steadily accumulating Pho1 enzyme during the interval from 1 to 6 h poststarvation (Fig. 12A). *spx1*Δ cells, which have a lower basal level of Pho1 expression, experienced a prolonged lag phase of about 3 h prior to the onset of Pho1 accumulation, after which activity increased with virtually the same slope as seen in wild-type cells (Fig. 12A). Comparison of the two kinetic profiles indicates that *spx1*Δ elicited a 2-h delay in the phosphate starvation response. A similar delay in the onset of induction of the *PHO* gene *tgp1* occurs when 5′-proximal poly(A) signals in the *nc-tgp1* lncRNA are mutated (11), from which it was surmised that precocious lncRNA termination contributes to an early phase of starvation-induced *PHO* gene derepression in which the lncRNA promoter remains active. The starvation response is then fully consolidated by turning off the interfering lncRNA promoters. By extension, the *spx1*Δ delay in the starvation response might reflect a requirement for Spx1 during the early transition to precocious lncRNA termination.

**FIG 13:**
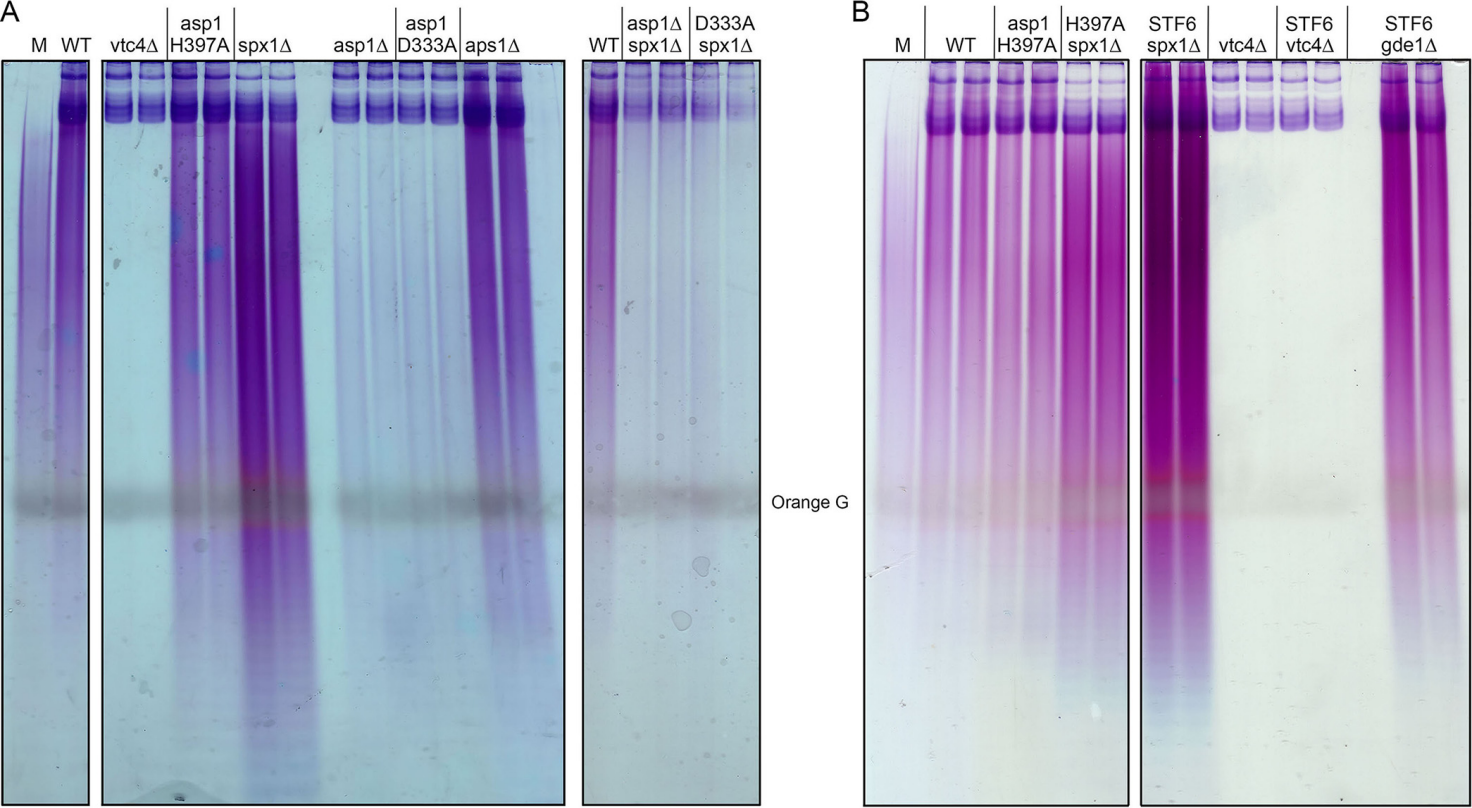
Effect of Spx1, Vtc4, Asp1, and Aps1 mutations on cellular polyphosphate content. Total polyphosphate prepared from the indicated fission yeast strains was analyzed by PAGE and visualized by staining with toluidine blue. The position of the Orange G dye marker is indicated.

A caveat to this idea ensues from a recent report from the Takeda lab in which they identified Spx1 (which they named Pqr1) as necessary to maintain the viability of fission yeast during quiescence induced by nitrogen starvation (35). They noted that the total cellular phosphate content of nitrogen-starved *pqr1*Δ mutant yeasts was 4-fold greater than that of a nitrogen-starved wild-type strain and suggested that hyperaccumulation of intracellular phosphate underlies the shortened chronological life span of nitrogen starved *pqr1*Δ cells. In support of that proposal, they found that omission of phosphate from the medium extended the life span of nitrogen-starved *pqr1*Δ cells. The higher total phosphate content seen in the absence of Pqr1/Spx1 was largely attributable to an increase in the intracellular content of inorganic polyphosphate synthesized by VTC (35). Here, we employed an optimized protocol to recover polyphosphates from whole-cell extracts (36), which were analyzed by polyacrylamide gel electrophoresis and stained with toluidine blue to gauge the polyphosphate content of phosphate-replete wild-type, *spx1*Δ, and *vtc4*Δ cells. We thereby affirmed that polyphosphate levels are higher in the absence of Spx1 and undetectable in the absence of Vtc4 (Fig. 13A and B). (The residual toluidine blue staining material near the top of the gel in the *vtc4*Δ sample is RNA.) Thus, it is possible that the delay we see in the phosphate starvation response in *spx1*Δ cells reflects a higher starting level of vacuolar polyphosphate, which can be catabolized to intracellular phosphate, so that it takes longer to deplete the phosphate pool to the point at which *PHO* gene transcription is derepressed.

A corollary to this line of thought is that ablating the intracellular pool of inorganic polyphosphate ought to accelerate or potentiate the phosphate starvation response by removing a buffer to cellular phosphate depletion. Indeed, we saw that the kinetics of Pho1 induction were hastened by about an hour in *vtc4*Δ and *vtc2*Δ cells compared to wild-type cells, whereby Pho1 activity in the *vtc4*Δ and *vtc2*Δ mutants was higher than that in wild-type cells at every time point sampled from 2 to 6 h poststarvation (Fig. 12B). We reasoned that if the sluggish starvation response in *spx1*Δ cells reflected a larger starting pool of polyphosphate, then the delay in Pho1 induction should be abolished by mutation of VTC. To test this, we constructed a *spx1*Δ *vtc2*Δ double mutant and found that its starvation response was also hastened compared to that of wild-type cells (Fig. 12B).

### Lack of correlation between total polyphosphate levels and suppression of IPP toxicosis.

Pascual-Ortiz et al. reported recently that cellular polyphosphate levels correlate with the levels of IP8 synthesized by Asp1 (37). In agreement with their findings, we see that the polyphosphate content of *asp1*Δ and *asp1-D333A* cells, which fail to synthesize IP8 and have elevated levels of IP7, is clearly reduced compared to that of the wild-type, albeit not depleted completely as in *vtc4*Δ cells (Fig. 13A). The polyphosphate content of the IPP pyrophosphatase-defective *aps1*Δ strain appears slightly higher than that of the wild-type (especially the population of shorter polymers running at the same rate as or faster than the Orange G dye), though not as elevated as in the *spx1*Δ strain (Fig. 13A). Note that Aps1-type IPP pyrophosphatase enzymes are also active as endopolyphosphatases (38, 39), raising the possibility that the increase in polyphosphate in *aps1*Δ cells reflects a contribution of Aps1 to polyphosphate turnover. The salient finding here is that the increase in polyphosphate content in the *spx1*Δ strain is eliminated in *asp1*Δ *spx1*Δ and *asp1-D333A spx1*Δ double mutants, where the polyphosphate levels mirror those of the *asp1*Δ and *asp1-D333A* single mutants (Fig. 13A). We surmise that IP8 activation of the VTC complex is necessary to achieve the increased polyphosphate content in *spx1*Δ cells.

*asp1-STF6 spx1*Δ cells accumulate markedly elevated levels of polyphosphate compared to wild-type and *asp1-H397A spx1*Δ cells (Fig. 13B). In contrast, *asp1-STF6 vtc4*Δ cells have no detectable polyphosphate (Fig. 13B). It is apparent from these data that the suppression of the lethal IPP pyrophosphatase mutation *asp1-STF6* by *spx1*Δ and *vtc4*Δ cannot be correlated with their diametrically opposite impact on total cellular polyphosphate content. Note that *asp1-STF6 gde1*Δ cells also have elevated polyphosphate content relative to the wild-type (Fig. 13B).

## DISCUSSION

The lethality of the *STF* alleles of Asp1 that truncate the IPP pyrophosphatase domain while preserving the kinase domain, and of the *asp1-H397A aps1*Δ double mutant that inactivates two IPP pyrophosphatase enzymes, is exerted via the agonistic effects of too much IP8 on RNA 3′-processing and transcription termination (12, 19). In principle, we can envision three ways to genetically ameliorate IPP toxicity in those IPP pyrophosphatase mutant strains: (i) by mutations of Pol2 or components of the 3′-processing/termination machinery that dampen the impact of toxic IP8 levels on Pol2 termination, (ii) by mutations that limit IPP synthesis or accelerate IPP decay so as to blunt the increase in IP8 levels when Asp1 and Aps1 IPP pyrophosphatases are crippled, and (iii) by mutations in hypothetical intermediary factors that transduce IP8 signals to the Pol2 transcription and RNA processing apparatus.

The results of the present *SST* screen consolidate the case for 3′-processing/termination as the proximal cause of IPP toxicosis via the identification of a hypomorphic mutation of the essential Cft1 subunit of CPF as a suppressor of lethal Asp1 pyrophosphatase mutations. This result accords with prior findings that IPP toxicosis is suppressed by deletion or loss-of-function mutations of the five inessential CPF subunits (Ppn1, Swd22, Ssu72, Dis2, and Ctf1) and of the termination factor Rhn1 (12, 19). Although the *SST* screen is clearly not yet saturated, it is noteworthy that we did not recover suppressors in genes with an immediate connection to inositol pyrophosphate synthesis or decay, notwithstanding that a most obvious way to mask the effects of IPP pyrophosphatase mutations that increase IP8 would be via a mutation in the Asp1 kinase domain that abolishes IP8 synthesis. Expanding the *SST* screen might eventually yield such suppressor mutations.

The most instructive outcome of the *SST* screen was the identification of loss-of-function mutations of SPX domain proteins Spx1 and Gde1, which in turn inspired a survey of the other four fission yeast SPX proteins that implicated Vtc4 and Vtc2 as additional agents of IPP toxicosis. SPX domains are regarded as IPP sensors (4). We find that alanine mutations of the IPP-binding sites of Spx1 and Gde1 phenocopy *spx1*Δ and *gde1*Δ with respect to relief of IPP toxicity. These results allow us to envision a genetic scenario whereby IPP binding to Spx1 and Gde1 domain is necessary to exert IPP toxicity. This is not the case for Vtc4, insofar as the IPP-binding site mutation in its SPX domain does not interdict IPP toxicity. Because the SPX domains in Gde1, Spx1, and the VTC complex are fused to flanking domain modules with known or imputed enzymatic activity, we queried the contributions of those activities to IPP toxicosis by introducing alanine mutations in the Gde1 and Vtc4 active sites and the Spx1 RING module. We thereby found that the Gde1 glycerophosphodiesterase, Vtc4 polyphosphate polymerase, and Spx1 RING ubiquitin ligase were necessary for IPP toxicosis. It is established that IPPs bound to the SPX domain are potent allosteric activators of the polyphosphate synthesis by the yeast VTC complex (34) and that fission yeast polyphosphate levels increase in IPP pyrophosphatase-defective *asp1-H397A* cells that have elevated levels of IP8 (37). We envision that IPP binding to the SPX domains of Gde1 and Spx1 regulates their enzymatic activities as well. The salient questions are how Gde1, Spx1, and VTC enable IPP toxicosis.

The enzymatic activity of Gde1 mobilizes inositol and inositol phosphates from phosphatidyl inositol and phosphoinositides, thereby providing upstream precursors for the synthesis of IP6 and, ultimately, IPPs. A parsimonious explanation for why loss of Gde1, or its catalytic activity, suppresses IPP toxicosis is that it reduces the available precursor pool for IP8 generation and thereby the impact of IPP pyrophosphatase loss-of-function mutations.

At this stage, any accounting for how polyphosphate synthesis by the VTC complex contributes to IPP toxicosis is highly speculative. Studies in budding yeast indicate that forced accumulation of nonphysiological levels of polyphosphate outside the vacuole (achieved via expression of a bacterial polyphosphate kinase in yeast) is *per se* cytotoxic (31). Coupling of VTC-mediated polyphosphate polymerase activity to vacuolar import of the polyphosphate product is seen as a means to avoid such toxicity (31). However, there is evidence that (i) vacuolar polyphosphate constitutes most but not all (∼80%) of the total polyphosphate in budding yeast, (ii) there is a pool of nuclear polyphosphate (dependent on Vtc4), and (iii) the nuclear polyphosphate pool persists in yeast cells engineered so that the intravacuolar pool of polyphosphate is depleted (40, 41). Indeed, a population of the Vtc2-containing VTC complex is localized around the nucleus in phosphate-replete cells (31). Thus, one can speculate that extraphysiological levels of IP8 that stimulate Vtc4 polyphosphate polymerase activity in IPP pyrophosphatase-dead mutants might promote the accumulation of polyphosphate in the fission yeast nucleus.

Polyphosphate can exert effects on cell physiology via nonenzymatic lysine pyrophosphorylation of target proteins *in vivo*, including nuclear proteins such as DNA topoisomerase I, Nsr1, and ribosome biogenesis factors (40–42). If lysine pyrophosphorylation contributes to IPP toxicosis (e.g., by modification of a component of the transcription apparatus), then it is expected that Vtc2- and Vtc4-null mutants would ameliorate the toxicity by eliminating the available nuclear polyphosphate pool. This scenario is consistent with our finding that a polyphosphate polymerase active site mutation R262A-R264A in Vtc4 that eliminates polyphosphate accumulation in fission yeast (Fig. S6) can suppress IPP toxicosis. In contrast, mutating the IPP-binding site in the Vtc4 SPX domain did not rescue IPP toxicity and only slightly reduced total polyphosphate content versus that in the wild-type (Fig. S6). It is noteworthy that mutating the Vtc4 SPX domain had less impact on polyphosphate content than did prevention of IP8 synthesis via deletion of Asp1 (compare Fig. S6 and Fig. 13A). A previous study documenting the importance of IPP binding to the VTC complex for polyphosphate synthesis by isolated yeast vacuoles was based on the effects of simultaneously mutating the IPP-binding residues of both SPX domains of the VTC complex (4). The present findings suggest that IPP binding to the SPX domain of fission yeast Vtc2 suffices for activation of the Vtc4 polyphosphate polymerase *in vivo* in the absence of IPP binding to Vtc4.

10.1128/mbio.03476-21.6 FIG S6 Effect of Vtc4 mutations on cellular polyphosphate content. Total polyphosphate prepared from wild-type cells, a *vtc4-*(*Y22A-K26A-K30A*) strain mutated in the IPP-binding site of the Vtc4 SPX domain, and a *vtc4-*(*R262A-R264A*) strain mutated in the active site of the polyphosphate polymerase domain was analyzed by PAGE and visualized by staining with toluidine blue. Download FIG S6, JPG file, 0.1 MB. Copyright © 2022 Schwer et al. 2022 Schwer et al. https://creativecommons.org/licenses/by/4.0/ This content is distributed under the terms of the Creative Commons Attribution 4.0 International license.

It is apparent that loss of Spx1 (or targeted mutations of its SPX and RING modules) is far more potent in overcoming IPP toxicosis than loss of Gde1 or VTC, insofar as (i) *spx1*Δ is able to suppress the lethality of *asp1-H397A aps1*Δ, while *gde1*Δ and *vtc4*Δ are not; (ii) Spx1 mutations erase the derepression of Pho1 that is characteristic of IPP pyrophosphatase mutants, whereas Gde1 and VTC mutations do not; and (iii) *spx1*Δ strongly hyperrepresses Pho1 in otherwise wild-type cells, but Gde1 and VTC mutations do not. Moreover, *spx1*Δ reverses the derepression of Pho1 elicited by alanine substitutions at Pol2 CTD positions Ser5, Pro6, and Ser7 and by Seb1-G476S. Because IPP pyrophosphatase and the aforementioned CTD and Seb1 mutations exert their respective cytotoxicity and/or derepression of the *PHO* genes via overzealous or precocious 3′-processing and transcription termination, it suggests that Spx1 might function in transducing an IP8-driven signal to the transcription and processing machinery.

In support of a transcriptional role for Spx1, the RNA-seq analysis of *spx1*Δ cells shows that the hyperrepression of *pho1*, *pho84*, and *tgp1* mRNAs is accompanied by an increase in the levels of read-through of the *PHO*-interfering lncRNAs *prt*, *prt*2, and *nc-tgp1* into the adjacent *PHO* mRNA transcription units. We can speculate that IP8 activates the Spx1 RING domain, leading to ubiquitylation of a component of the transcription/processing machinery that results in precocious termination and that this effect would be tunable by cellular IP8 levels. Absence of such ubiquitylation in *spx1*Δ cells would result in less efficient 3′-processing/termination that suffices to overcome the agonistic effects of IPP pyrophosphatase, CTD, and Seb1 mutations.

In an alternative scenario, Spx1 could be a positive regulator of IP8 synthesis (e.g., via an activating ubiquitylation of the Asp1 kinase domain or an inactivating ubiquitylation of a hypothetical inhibitor of IPP synthesis) such that *spx1*Δ cells would be deficient in IP8 synthesis. Although *spx1*Δ cells resemble Asp1 kinase null (*asp1*Δ) or Asp1 kinase-dead (*asp1-D333A*) mutants with respect to hyperrepression of the *PHO* genes and attenuation of Pho1 derepression by *CTD-S7A* (12) and *seb1-G476S* (30), the Spx1-null and Asp1 kinase mutant phenotypes diverge in ways that argue against attributing *spx1*Δ suppression of IPP toxicosis to a deficiency of IP8. Specifically, (i) lack of IP8 in *asp1*Δ cells results in depletion of the cellular pool of inorganic polyphosphates synthesized by the IP8-dependent VTC complex, whereas lack of Spx1 leads to an increase in cellular polyphosphate, and (ii) *asp1*Δ is synthetically lethal with CPF *ppn1*Δ, *swd22*Δ, and *ssu72-C13S* loss-of-function mutants (12), whereas *spx1*Δ *ppn1*Δ, *spx1*Δ *swd22*Δ, and *spx1*Δ *ssu72-C13S* double mutants are viable (Fig. S7). Although it appears, on the surface, contradictory that IPP toxicity is ameliorated by *spx1*Δ, which increases total polyphosphate content, and by *vtc4*Δ, which eliminates polyphosphate production, the conundrum can be rationalized if the increase in *spx1*Δ cells is confined to the vacuole, where it can serve as a source of mobilizable phosphate during acute phosphate starvation, thereby accounting for the observed delay in *pho1* induction in phosphate-starved *spx1*Δ cells.

10.1128/mbio.03476-21.7 FIG S7 Lack of synthetic growth defects in *spx1*Δ *CPF/rhn1* double mutants. The indicated fission yeast strains were spot tested for growth on YES agar at 30°C. Download FIG S7, TIF file, 1.1 MB. Copyright © 2022 Schwer et al. 2022 Schwer et al. https://creativecommons.org/licenses/by/4.0/ This content is distributed under the terms of the Creative Commons Attribution 4.0 International license.

## MATERIALS AND METHODS

### Spot tests of fission yeast growth.

Cultures of S. pombe strains were grown in liquid YES (yeast extract with supplement) medium until *A*_600_ reached 0.5 to 0.8. The cultures were adjusted to an *A*_600_ of 0.1, and aliquots (3 μL) of serial 5-fold dilutions were spotted to YES agar. The plates were photographed after incubation for 2 days at 34°C, 2.5 days at 30°C and 37°C, 4 days at 25°C, and 6 days at 20°C.

### Acid phosphatase activity.

Cells were grown at 30°C in YES medium. Aliquots of exponentially growing cultures were harvested, washed with water, and resuspended in water. To quantify acid phosphatase activity, reaction mixtures (200 μL) containing 100 mM sodium acetate (pH 4.2), 10 mM *p*-nitrophenylphosphate, and cells (ranging from 0.01 to 0.1 *A*_600_ unit) were incubated for 5 min at 30°C. The reactions were quenched by addition of 1 mL of 1 M sodium carbonate, the cells were removed by centrifugation, and the absorbance of the supernatant at 410 nm was measured. Acid phosphatase activity is expressed as the ratio of *A*_410_ (*p*-nitrophenol production) to *A*_600_ (cells). The data are averages (and standard error of the mean [SEM]) for at least three assays using cells from three independent cultures.

### Whole-genome sequencing and mapping suppressor mutations.

After PicoGreen quantification and quality control by Agilent Bioanalyzer, 500-ng aliquots of genomic DNA from the four *SST* strains were sheared using a LE220-plus focused ultrasonicator (Covaris catalog number 500569), and sequencing libraries were prepared using the KAPA Hyper Prep kit (Kapa Biosystems KK8504) with modifications. DNA libraries were subjected to size selection by mixture with 0.5 vol of AMPure XP beads (Beckman Coulter catalog number A63882) after postligation cleanup. Libraries were not amplified by PCR and were pooled equivolume for sequencing. Samples were run on a NovaSeq 6000 in a 150-bp/150-bp paired-end run using the NovaSeq 6000 SBS v1 kit and an S1 flow cell (Illumina). The average number of read pairs per sample was 10 million. The sequencing data from *SST* strains were aligned to the genome using Bowtie2 (43). The resulting SAM files were converted to BAM files using SAMtools (44). Variants were identified by comparing the parental *STF* genome (19) to the mutant *SST* genome using BCFtools (45) with the criteria of an adjusted mapping quality of 40, a minimum base quality of 20, and disabled probabilistic realignment for the computation of base alignment quality for considering variations or insertion-deletion events. The multiallelic caller protocol was used for variant calling in BCFtools. Variants were annotated using SnpEff, with its in-built genome version for S. pombe (46). Variants were further filtered by removing all variations with an average mapping quality ≤25 (Phred scale). All variants present in the parental strain were excluded as noncausal mutations.

### Gene deletions.

PCR amplification and standard cloning methods were employed to construct plasmids in which a *kanMX* cassette (47) is flanked by 460- to 830-bp gene-specific DNA segments corresponding to genomic sequences upstream and downstream of the ORF, thereby deleting *spx1* (*SPAC6B12.07c*) from nucleotides +1 to +1413 (relative to the translational start codon, +1), *gde1* from nucleotides +164 to +3418, *vtc2* from nucleotides +8 to +2412, *vtc4* from nucleotides +1 to +2178, *plt1* from nucleotides +1 to +2853, and *spx2*(*SPCC1827.07c*) from nucleotides +15 to +2427. The disruption cassettes were excised from the plasmids and transfected into diploid S. pombe cells. G418-resistant transformants were selected and analyzed by Southern blotting to confirm correct integration at one of the loci. Heterozygous diploids were sporulated and G418-resistant haploids were isolated. A nourseothricin-resistant *vtc2*Δ::*natMX* strain was generated by marker switching (48).

### Allelic replacements.

To generate strains harboring the marked wild-type and mutated *spx1*, *gde1*, or *vtc4* alleles, we first constructed Bluescript-based plasmids containing integration cassettes for each of the alleles, via PCR amplification and standard cloning methods. The integration cassette for wild-type *spx1* consists of four elements in series from 5′ to 3′: (i) a 2.16-kb DNA segment of genomic DNA spanning −700 to +1460 (relative to the translational start codon +1) of the *spx1* locus, (ii) a 268-bp segment including poly(A)/termination signals from the *nmt1^+^* gene, (iii) a *kanMX* gene conferring resistance to G418, and (iv) a 635-bp segment (+1460 to +2095) of genomic DNA 3′ of the *spx1^+^* ORF. In the integration cassette for wild-type *vtc4*, the *kanMX* gene is flanked 5′ by a 2.7-kb DNA segment of genomic DNA spanning −530 to +2248 (relative to the ATG at +1) of the *vtc4* locus and 3′ by a 726-bp segment (+2244 to +2970) of genomic DNA downstream of the *vtc4* ORF. The integration cassette for *gde1* consists of a 4.08-kb DNA segment of genomic DNA (−647 to +3433 relative to the Leu start codon at +1) of the *gde1*^+^ locus, followed by the *kanMX* gene and a 461-bp segment of genomic DNA 3′ of the *gde1*^+^ stop codon. Two-stage PCR overlap extension with mutagenic primers was used to introduce an ATG in lieu of the Leu codon in wild-type *gde1* and to introduce missense mutations into the *spx1*, *gde1*, and *vtc4* ORFs. Mutated DNA restriction fragments were then inserted into the integration cassettes in lieu of the respective wild-type ORFs. All inserts were sequenced to exclude the presence of unwanted mutations. The integration cassettes were excised from the plasmids and transfected into diploid S. pombe cells. G418-resistant transformants were selected and correct integrations at the target locus were confirmed by Southern blotting. A segment of the mutated *kanMX*-marked allele was amplified by PCR in each case and sequenced to verify that the desired mutations were present. The heterozygous diploids were then sporulated, and G418-resistant haploids were isolated.

### Tests of mutational synergies.

Standard genetic methods (49) were employed to generate haploid strains harboring mutations/deletions in two (or three) differently marked genes. In brief, pairs of haploids with null or missense mutations were mixed on malt agar to allow mating and sporulation, and the mixture was then subjected to random spore analysis. Spores (∼1,500) were plated on YES agar and also on medium selective for marked mutant alleles; the plates were incubated at 30°C for up to 5 days to allow slow-growing progeny to germinate and form colonies. At least 500 viable progeny were screened by replica plating for the presence of the second (and then third) marker gene, or by sequentially replica plating from YES to selective medium. A finding that no haploids with two marker genes were recovered after 6 to 8 days of incubation at 30°C was taken to indicate synthetic lethality. In cases where a strain was crossed to *STF6* or *STF9* cells bearing the *rpb1-T4A*::*natMX* allele, sequentially selected double mutants were then tested for nourseothricin resistance by replica plating to select those that had lost the *T4A* allele. A finding that all of the double mutants were nourseothricin resistant was taken to indicate lack of suppression. Growth phenotypes of viable double- and triple-mutant strains were assessed in parallel with those of the parental and wild-type cells at different temperatures (20°C to 37°C) by spotting as described above.

### Transcriptome profiling by RNA-seq.

RNA was isolated from S. pombe *spx1*^+^ and *spx1*Δ cells that were grown in liquid YES medium at 30°C to an *A*_600_ of 0.5 to 0.6. Cells were harvested by centrifugation, and total RNA was extracted via the hot phenol method. The integrity of total RNA was gauged with an Agilent Technologies 2100 Bioanalyzer. The Illumina TruSeq stranded mRNA sample preparation kit was used to purify poly(A)^+^ RNA from 500 ng of total RNA and to carry out the subsequent steps of poly(A)^+^ RNA fragmentation, strand-specific cDNA synthesis, indexing, and amplification. Indexed libraries were normalized and pooled for paired-end sequencing performed by using an Illumina NovaSeq 6000-S1 flow cell. FASTQ files bearing paired-end reads of 51 bases (total paired reads of 14.7 million to 30.1 million per biological replicate) were mapped to the S. pombe genome (Pombase) using HISAT2-2.1.0 with default parameters (50). The resulting SAM files were converted to BAM files using SAMtools (44). Count files for individual replicates were generated with HTSeq-0.10.0 (51) using exon annotations from Pombase (GFF annotations, genome-version ASM294v2; source, ensembl). RPKM (reads per kilobase per million) analysis and pairwise correlations (Pearson coefficients of 0.953 to 0.986) were performed as described previously (52). Differential gene expression and fold change analysis was performed in DESeq2 (53). Cutoff for further evaluation was set for genes that had an adjusted *P* value (Benjamini-Hochberg corrected) of ≤0.05 and were up or down by at least 2-fold in *spx1*Δ versus the wild-type. Genes were further filtered on the following criteria: (i) ≥2-fold up and an average normalized read count for the mutant strain of ≥100 and (ii) ≥2-fold down and an average normalized read count for the wild-type strain of ≥100.

### Phosphate starvation response.

Fission yeast strains were grown at 30°C in YES medium to an *A*_600_ of 0.5 to 0.8. The cells were harvested, washed with water, and adjusted to an *A*_600_ of ∼0.3 in PMG (pombe minimal glutamate) medium without phosphate after withdrawing an aliquot to measure Pho1 activity (time zero). Acid phosphatase Pho1 activity was assayed every hour during a 6-h period of phosphate starvation.

### PAGE assay of intracellular polyphosphate content.

S. pombe cells were grown in YES medium at 30°C. Aliquots (5 *A*_600_ units; ∼8 × 10^8^ cells) of logarithmically growing cultures were harvested by centrifugation, washed with cold water, and stored at −80°C. The cell pellets were resuspended in 400 μL AE buffer (50 mM sodium acetate [pH 5.2], 10 mM EDTA) and added to 300 μL phenol (equilibrated in 10 mM Tris-HCl [pH 8.0]) plus 40 μL of 10% (wt/vol) SDS. The samples were mixed vigorously and incubated at 65°C for 5 min and then placed on ice. Chloroform (300 μL) was added, and the mixed samples were centrifuged for 5 min at room temperature at 17,000 × *g*. The aqueous phase (∼450 μL) was collected, extracted with phenol-chloroform and chloroform, and then ethanol precipitated at −20°C overnight. The precipitates were washed with 70% ethanol, dried, and resuspended in 75 μL TE (10 mM Tris-HCl [pH 7.0], 1 mM EDTA; 15 μL per 1 *A*_600_ unit of cells). Aliquots (10 μL) were supplemented with 3× Orange G dye (10 mM Tris-HCl [pH 7.0], 1 mM EDTA, 30% glycerol, 0.1% [wt/vol] Orange G) and then analyzed by electrophoresis through a 20% polyacrylamide gel in TBE (80 mM Tris-borate, 1 mM EDTA) at 4°C for 16 to 18 h at 3 mA. The gels were stained with toluidine blue (0.05% [wt/vol] toluidine blue in 20% methanol, 2% glycerol) and scanned after destaining.

### Data availability.

The RNA-seq data in this publication have been deposited in NCBI's Gene Expression Omnibus and are accessible through GEO Series accession number GSE185441.

10.1128/mbio.03476-21.9 TABLE S2 List of fission yeast strains constructed for this study. Download Table S2, DOCX file, 0.04 MB. Copyright © 2022 Schwer et al. 2022 Schwer et al. https://creativecommons.org/licenses/by/4.0/ This content is distributed under the terms of the Creative Commons Attribution 4.0 International license.
