# Supplementary figures and images for: Cleavage-Polyadenylation Factor Cft1 and SPX Domain Proteins Are Agents of Inositol Pyrophosphate Toxicosis in Fission Yeast
Source: mBio. 2022 Jan 11;13(1):e03476-21. doi: 10.1128/mbio.03476-21 (PMC8749416; doi:10.1128/mbio.03476-21)

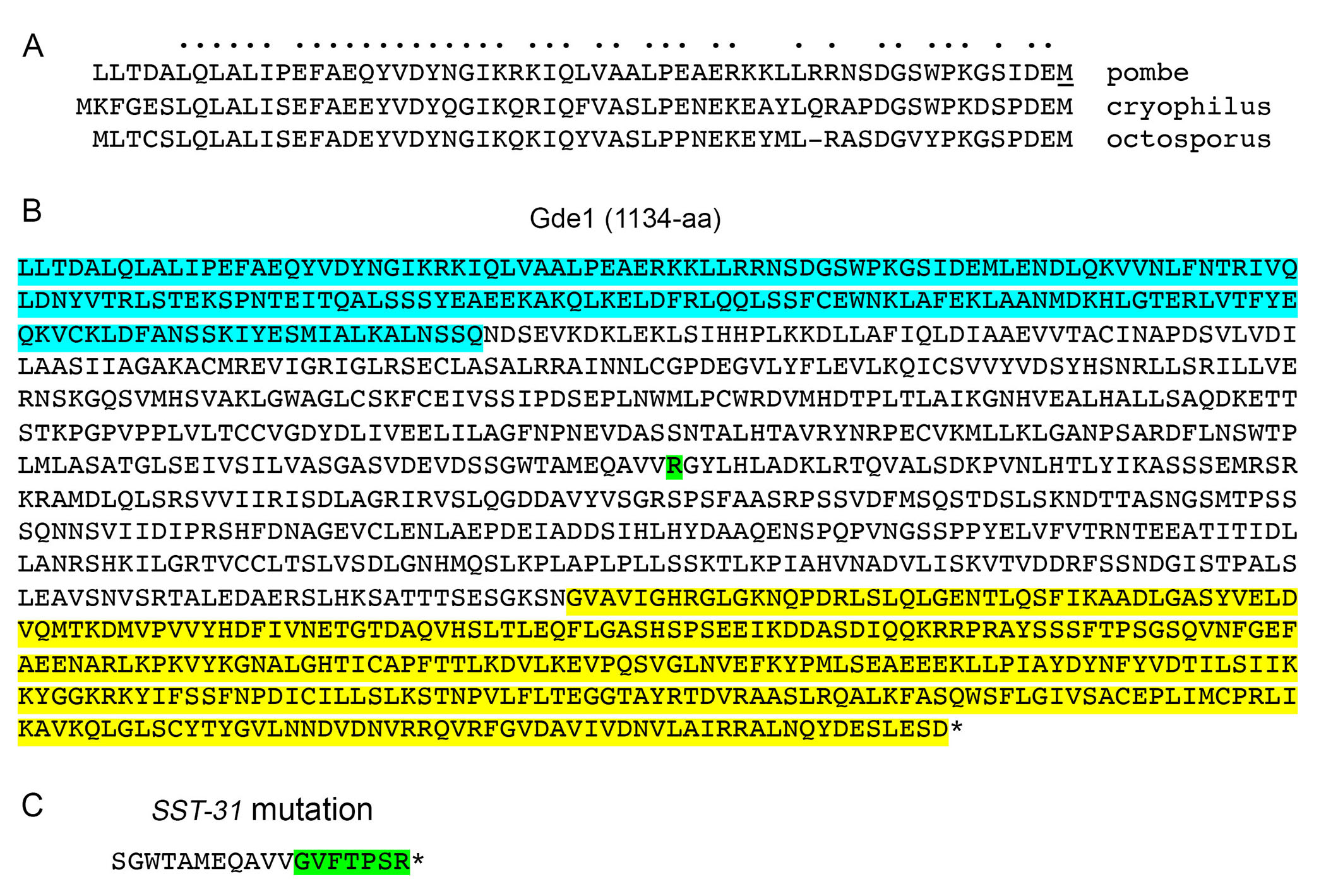

Supplement: FIG S1 [file mbio.03476-21-sf001.jpg]

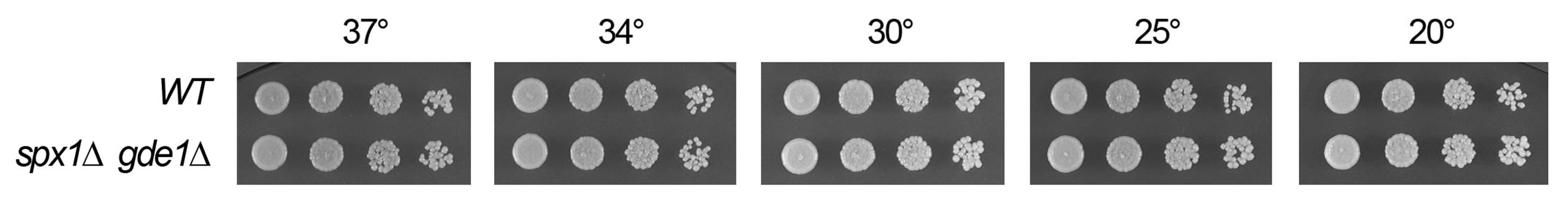

Supplement: FIG S2 [file mbio.03476-21-sf002.tif]

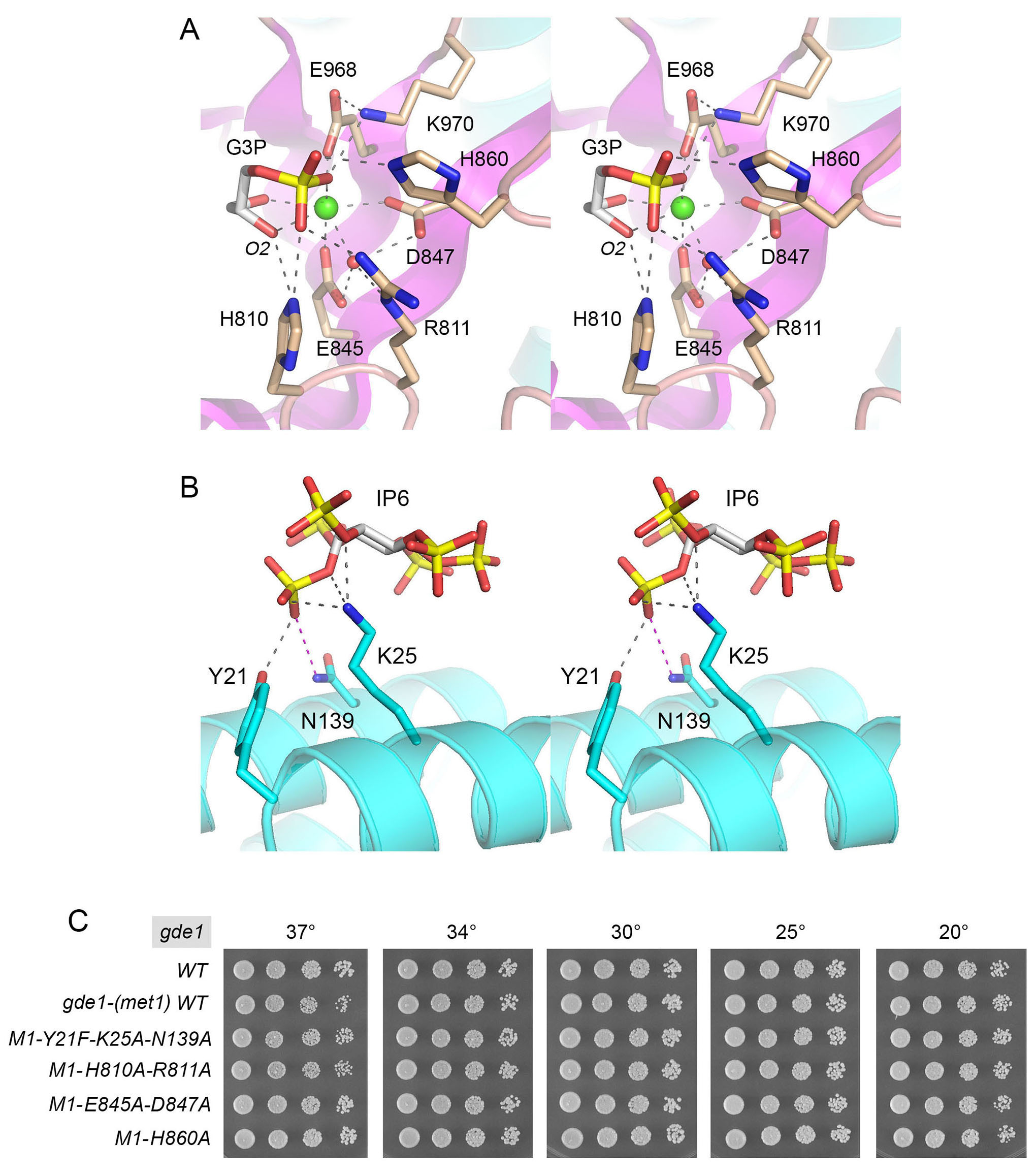

Supplement: FIG S3 [file mbio.03476-21-sf003.jpg]

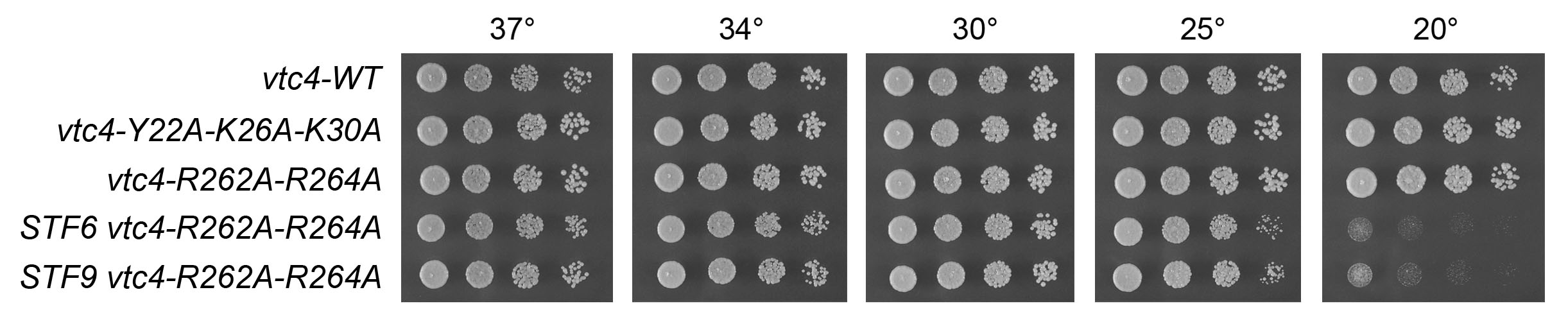

Supplement: FIG S4 [file mbio.03476-21-sf004.tif]

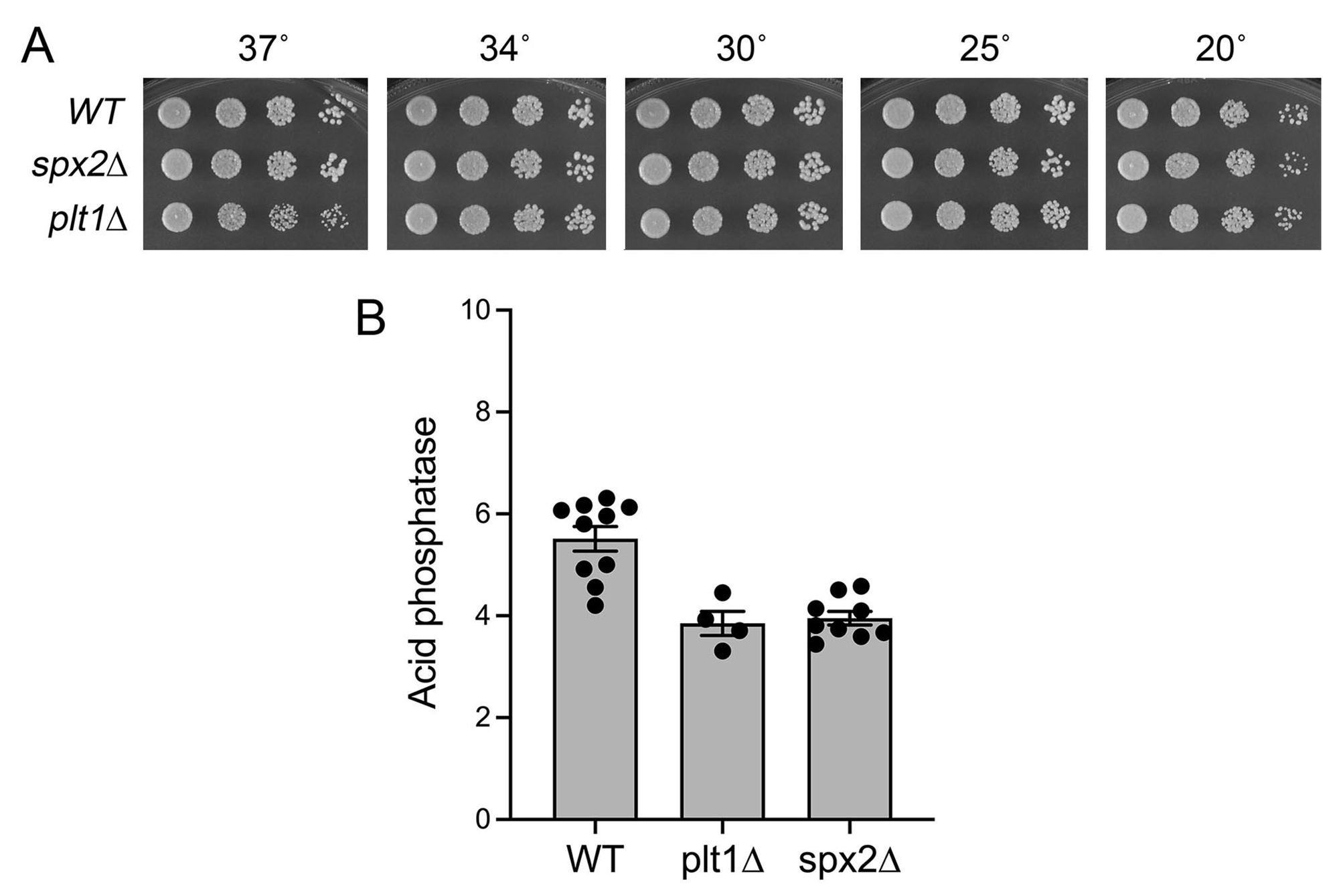

Supplement: FIG S5 [file mbio.03476-21-sf005.tif]

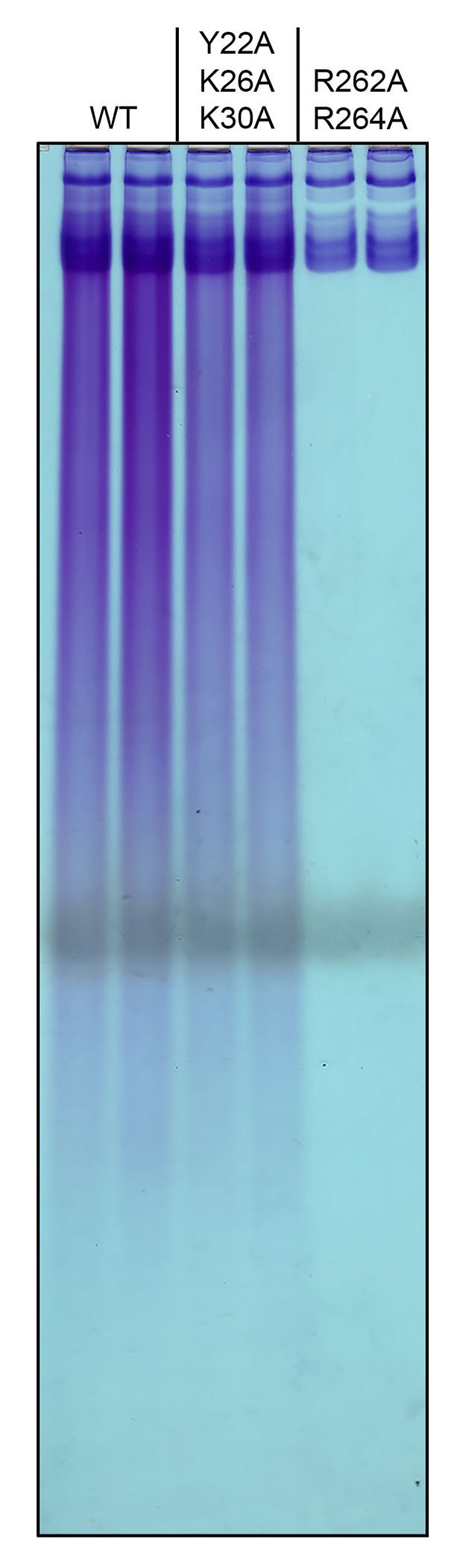

Supplement: FIG S6 [file mbio.03476-21-sf006.jpg]

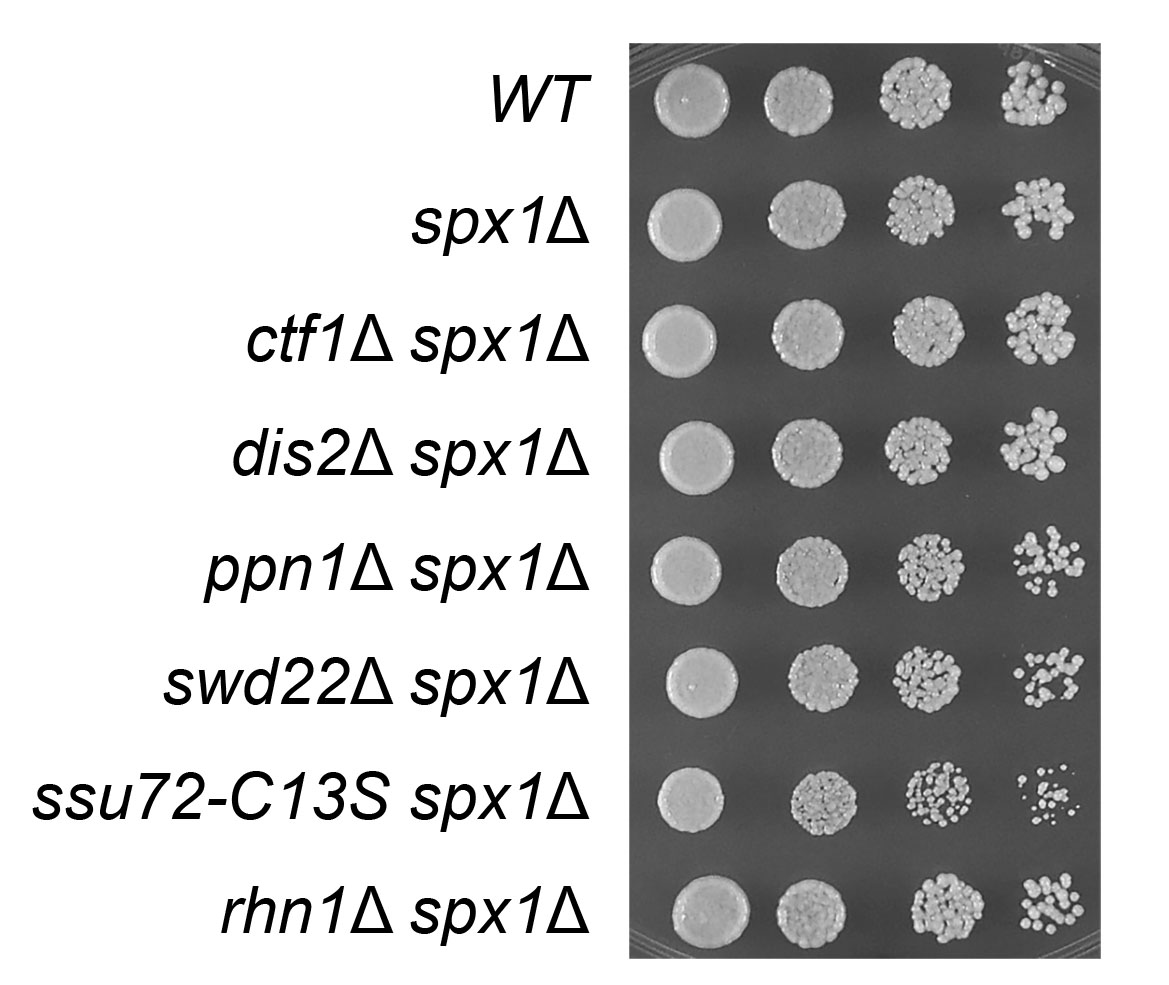

Supplement: FIG S7 [file mbio.03476-21-sf007.tif]
