# Supplementary material for: Cleavage-Polyadenylation Factor Cft1 and SPX Domain Proteins Are Agents of Inositol Pyrophosphate Toxicosis in Fission Yeast
Source: mBio. 2022 Jan 11;13(1):e03476-21. doi: 10.1128/mbio.03476-21 (PMC8749416; doi:10.1128/mbio.03476-21)
Supplement: TABLE S2 [file mbio.03476-21-st002.docx]

Table S2. List of fission yeast strains constructed for this study.

| **Strain** | **Genotype** |
| --- | --- |
| BS246 | *SST-31 STF-3 [asp1-G863D] rpb1-WT::kanMX* |
| BS249 | *SST-52 STF-5 [asp1-C643Y] rpb1-WT::kanMX* |
| BS250 | *SST-53 STF-5 [asp1-C643Y] grt1-S223R prp43-D25E rpb1-WT::kanMX* |
| BS255 | *SST-75 STF7 [asp1-H686Y] rpb1-WT::kanMX* |
| BS53 | *h+* *spx1∆::kanMX* |
| BS78 | *h+* *vtc2∆::kanMX* |
| BS127 | *h*+ *vtc4∆::kanMX* |
| BS129 | *h- gde1∆::kanMX* |
| BS81 | *h- plt1∆::natMX* |
| AS1842 | *h- spx2∆::kanMX* |
| BS497 | *h+ spx1∆::ura4MX* |
| BS80 | *h+ vtc2*∆*::natMX* |
| BS164 | *h+ spx1∆::kanMX vtc2∆::natMX* |
| BS511 | *h+ gde1∆::kanMX spx1∆::ura4MX* |
| BS352 | *h- spx1∆::kanMX STF6::hygMX [asp1-W386*]* |
| BS353 | *h- spx1∆::kanMX STF9::hygMX [asp1-W493*]* |
| BS354 | *h- spx1∆::kanMX asp1-H397A::natMX* |
| BS401 | *h+ spx1∆::kanMX aps1∆::hygMX* |
| BS355 | *h+ spx1∆::kanMX asp1∆::natMX* |
| BS356 | *h+ spx1∆::kanMX asp1-D333A::natMX* |
| BS358 | *h- gde1∆::kanMX STF6::hygMX [asp1-W386*]* |
| BS359 | *h- gde1∆::kanMX STF9::hygMX [asp1-W493*]* |
| BS360 | *h+ gde1∆::kanMX asp1-H397A::natMX* |
| BS362 | *h+ gde1∆::kanMX asp1-D333A::natMX* |
| BS372 | *h- SST-75 [cft1-C823R] STF6::hygMX [asp1-W386*]* |
| BS373 | *h- SST-75 [cft1-C823R] STF9::hygMX [asp1-W493*]* |
| BS400 | *h+ spx1::kanMX asp1-H397A::natMX aps1*∆*::hygMX* |
| BS402 | *h+ vtc4∆::kanMX STF6::hygMX [asp1-W386*]* |
| BS403 | *h- vtc4∆::kanMX STF9::hygMX [asp1-W493*]* |
| BS415 | *h- vtc2∆::kanMX STF6::hygMX [asp1-W386*]* |
| BS416 | *h- vtc2∆::kanMX STF9::hygMX [asp1-W493*]* |
| BS475 | *h+ spx1-WT::kanMX* |
| BS477 | *h+ spx1-Y26A-K30A-K34A::kanMX* |
| BS479 | *h+ spx1-C374A-C377A::kanMX* |
| BS481 | *h- spx1-C394A-C397A::kanMX* |
| BS482 | *h+ spx1-C409A-C412A::kanMX* |
| BS484 | *h- spx1∆::kanMX ctf1∆::ura4+* |
| BS485 | *h+ spx1∆::kanMX dis2∆::ura4+* |
| BS486 | *h- spx1∆::kanMX ppn1∆::hygMX* |
| BS487 | *h+ spx1∆::kanMX swd22∆::hygMX* |
| BS488 | *h+ spx1∆::kanMX rhn1∆::hygMX* |
| BS357 | *h+ spx1∆::kanMX ssu72-C13S::natMX* |
| BS489 | *h- spx1-Y26A-K30A-K34A::kanMX STF6::hygMX [asp1-W386*]* |
| BS490 | *h- spx1-C374A-C377A::kanMX STF6::hygMX [asp1-W386*]* |
| BS491 | *h- spx1-C394A-C397A::kanMX STF6::hygMX [asp1-W386*]* |
| BS492 | *h- spx1-C409A-C412A::kanMX STF6::hygMX [asp1-W386*]* |
| BS493 | *h- spx1-Y26A-K30A-K34A::kanMX STF9::hygMX [asp1-W493*]* |
| BS494 | *h- spx1-C374A-C377A::kanMX STF9::hygMX [asp1-W493*]* |
| BS495 | *h- spx1-C394A-C397A::kanMX STF9::hygMX [asp1-W493*]* |
| BS496 | *h- spx1-C409A-C412A::kanMX STF9::hygMX [asp1-W493*]* |
| BS542 | *h+ gde1-WT(met)::kanMX* |
| BS550 | *h+ gde1-(met)-Y21F-K25A-N139A::kanMX* |
| BS554 | *h+ gde1-(met)-H810A-R811A::kanMX* |
| BS558 | *h+ gde1-(met)-E845A-D847A::kanMX* |
| BS562 | *h+ gde1-(met)-H860A::kanMX* |
| BS566 | *h- spx1∆::kanMX rpb1-WT::natMX* |
| BS567 | *h- spx1∆::kanMX rpb1-S7AT::natMX* |
| BS569 | *h- spx1∆::kanMX rpb1-S5•S5A::natMX* |
| BS570 | *h- spx1∆::kanMX rpb1-P6•P6A::natMX* |
| BS586 | *h- gde1-(met)-Y21F-K25A-N139A::kanMX STF6::hygMX [asp1-W386*]* |
| BS587 | *h- gde1-(met)-H810A-R811A::kanMX STF6::hygMX [asp1-W386*]* |
| BS588 | *h- gde1-(met)-E845A-D847A::kanMX STF6::hygMX [asp1-W386*]* |
| BS589 | *h- gde1-(met)-H860A::kanMX STF6::hygMX [asp1-W386*]* |
| BS590 | *h+ gde1-(met)-Y21F-K25A-N139A::kanMX STF9::hygMX [asp1-W493*]* |
| BS591 | *h+ gde1-(met)-H810A-R811A::kanMX STF9::hygMX [asp1-W493*]* |
| BS592 | *h- gde1-(met)-E845A-D847A::kanMX STF9::hygMX [asp1-W493*]* |
| BS593 | *h- gde1-(met)-H860A::kanMX STF9::hygMX [asp1-W493*]* |
| BS594 | *h+ vtc2∆::kanMX asp1-H397A::hygMX* |
| BS595 | *h+ vtc4∆::kanMX asp1-H397A::hygMX* |
| BS615 | *h- spx1∆::kanMX seb1-G476S::hygMX* |
| BS618 | *h- vtc4-WT::kanMX* |
| BS620 | *h- vtc4-Y22A-K26A-K30A::kanMX* |
| BS622 | *h- vtc4-R262A-R264A::kanMX* |
| BS624 | *h- vtc4-R262A-R264A::kanMX STF6::hygMX [asp1-W386*]* |
| BS625 | *h- vtc4-R262A-R264A::kanMX STF9::hygMX [asp1-W493*]* |

All strains are *leu1-32* *ura4-D18* *his3-D1* and either *ade6-m216 or ade6-m210*. The *asp1* alleles present in the *STF* mutants are specified in brackets *[ ]*.
